# Practical Approach to Histological Diagnosis of Peripheral Nerve Sheath Tumors: An Update

**DOI:** 10.3390/diagnostics12061463

**Published:** 2022-06-14

**Authors:** Gaetano Magro, Giuseppe Broggi, Giuseppe Angelico, Lidia Puzzo, Giada Maria Vecchio, Valentina Virzì, Lucia Salvatorelli, Martino Ruggieri

**Affiliations:** 1 Anatomic Pathology, Department of Medical, Surgical Sciences and Advanced Technologies “G.F. Ingrassia”, University of Catania, 95123 Catania, Italy; giuseppe.broggi@gmail.com (G.B.); lipuzzo@unict.it (L.P.); giadamariavecchio@gmail.com (G.M.V.); lucia.salvatorelli@unict.it (L.S.); 2 Pathology Unit, Cannizzaro Hospital, 95126 Catania, Italy; giuangel86@hotmail.it; 3 Unit of Diagnostic Radiology, Regina Pacis Clinic, 93017 San Cataldo, Italy; valentinavirzi@gmail.com; 4 Unit of Rare Diseases of the Nervous System in Childhood, Department of Clinical and Experimental Medicine, University of Catania, 95124 Catania, Italy; m.ruggieri@unict.it

**Keywords:** peripheral nerve sheath tumors, neurofibroma, schwannoma, perineurioma, neurofibromatosis type 1, neurofibromatosis type 2, schwannomatosis

## Abstract

Peripheral nerve sheath tumors encompass a wide spectrum of lesions with different biological behavior, including both benign and malignant neoplasms as well as the recent diagnostic category, i.e., “*atypical neurofibromatous neoplasm with uncertain biologic potential*” to be used only for NF1 patients. Neurofibromas and schwannomas are benign Schwann-cell-derived peripheral nerve sheath tumors arising as isolated lesions or within the context of classical neurofibromatosis or schwannomatoses. Multiple tumors are a hallmark of neurofibromatosis type 1(NF1) and related forms, NF2-related-schwannomatosis (formerly NF2) or SMARCB1/LZTR1-related schwannomatoses. Perineuriomas are benign, mostly sporadic, peripheral nerve sheath tumors that show morphological, immunohistochemical, and ultrastructural features reminiscent of perineurial differentiation. Hybrid tumors exist, with the most common lesions represented by a variable mixture of neurofibromas, schwannomas, and perineuriomas. Conversely, malignant peripheral nerve sheath tumors are soft tissue sarcomas that may arise from a peripheral nerve or a pre-existing neurofibroma, and in about 50% of cases, these tumors are associated with NF1. The present review emphasizes the main clinicopathologic features of each pathological entity, focusing on the diagnostic clues and unusual morphological variants.

## 1. Introduction

The interest for peripheral nerve sheath tumors is mainly due to the fact that these tumors can be diagnosed in the context of tumor-predisposing syndromes, such as neurofibromatosis type 1 (NF1) and related forms, NF2-related-schwannomatosis (formerly NF2) or SMARCB1/LZTR1-related schwannomatoses (formerly schwannomatosis) [1]. Based on recent clinical and molecular advances, the diagnostic criteria of the above-mentioned syndromes have been updated [2,3]. The present review focuses on the pathological diagnostic clues of the most common benign and malignant peripheral nerve sheath tumors to aid pathologists in achieving a correct classification. The main clinicopathologic features of each single pathological entity are discussed and summarized in tables. Representative illustrations, including the morphological variants of each single tumor are also provided. Awareness by surgical pathologists of the wide morphological spectrum of these tumors and their development in the context of tumor-predisposing syndromes is crucial for providing correct prognostic information and planning the clinical/radiological follow-up of patients.

## 2. Classification of the Peripheral Nerve Sheath Tumors

The current pathological classification of the peripheral nerve sheath tumors includes both benign and malignant tumors, with the recent addition of a diagnostic category, i.e., “*atypical neurofibromatous neoplasm with uncertain biologic potential (ANNUBP)*” to be used only for patients with NF1 [1,4,5,6,7] (Table 1).

## 3. Neurofibromas

**Figure 1:**
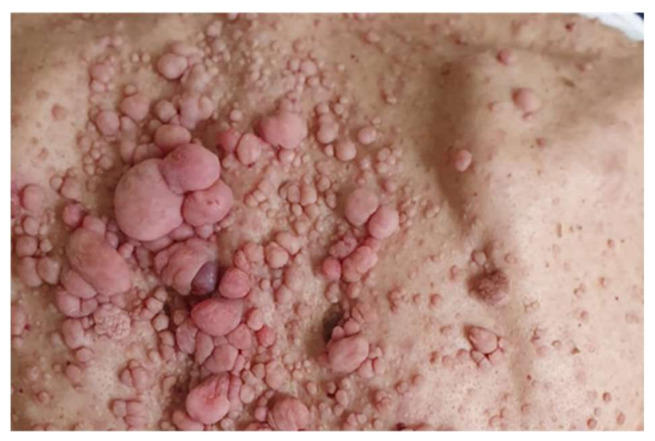
A 64-year-old male patient with NF1: multiple and variable-sized cutaneous neurofibromas of his back.

**Figure 2:**
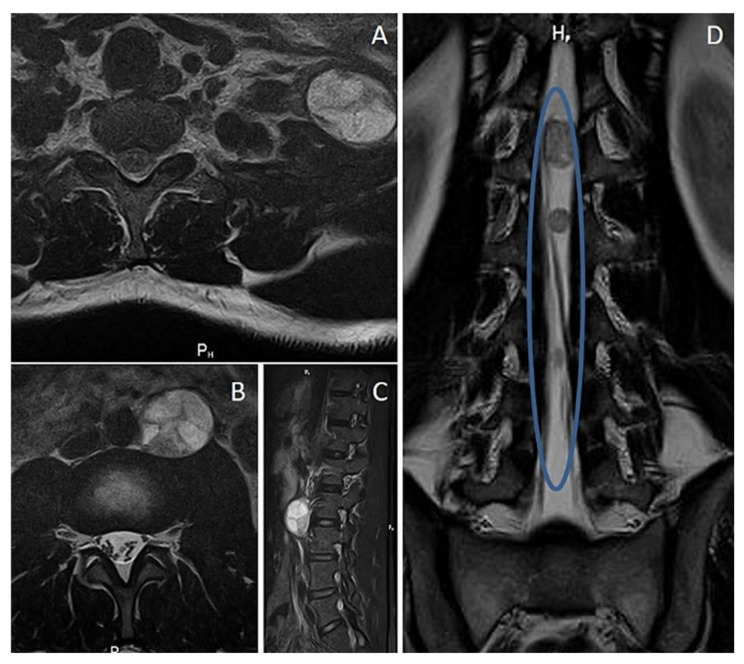
Multiple neurofibromas in an NF1 patient. (**A**) Left laterocervical mass, non-homogeneous, well-defined T2 axial view; (**B**) left lumbar para-aortic mass on T2 axial view and (**C**) STIR sagittal view. (**D**) Intracanalar lumbar tract myelography MRI.

Neurofibromas are benign peripheral nerve sheath tumors mainly composed of neoplastic cells showing schwannian differentiation admixed to a minor component of cells with fibroblastic and perineurial differentiation. They represent the most common peripheral nerve sheath tumors and are usually diagnosed as apparently sporadic lesions; however, there is increasing evidence that a large number of histologically proven neurofibromas arise within the context of classical neurofibromatosis type 1 (NF1) or its alternative forms (Table 2), and that, when isolated, they may be caused by mosaic phenomena occurred in the *NF1* gene at a somatic level, and it is currently accepted that virtually all individuals affected by NF1 develop neurofibromas (Figure 1 and Figure 2) [8]. Based on the recently revised criteria for NF1 [2] and a newly proposed classification of the different forms of neurofibromatosis and schwannomatoses [9,10,11] (Table 2), a surgical pathologist should be aware of the possibility of this spectrum of disorders when he/she is dealing with an isolated neurofibroma, with multiple lesions or a plexiform neurofibroma from an individual without a personal or familial history of classical NF1 (Table 2); similarly, the chance of being affected by classical NF1 is likely when dealing with the above-mentioned tumors arising in a child with an affected parent by NF1. Mosaic NF1 (Table 2) should be suspected when a surgical biopsy/specimens are referred to a pathologist as an isolated neurofibroma, a single plexiform neurofibroma or when multiple cutaneous and/or nodular neurofibromas were arranged in a clearly segmental/localised distribution. In all these circumstances, a surgical pathologist should always suggest a clinical evaluation and genetic counselling by an NF1 expert.

The majority of neurofibromas occur in the skin and arise from small-sized nerves. These tumors can develop in the deep soft tissues from a major or small-sized nerve [7]; however, there is the possibility that the anatomic association with a nerve cannot be demonstrated, and neurofibroma may present as a soft tissue mass. Notably, although neurofibromas may develop from spinal nerve roots, often as multiple lesions in NF1 patients or as lesions involving, bilaterally, all the spinal roots in individuals with spinal NF1 (Table 2), it is of note that the cranial nerves are spared [7]. Histologically, neurofibroma is a neoplasm with low to moderate cellularity, composed of bland-looking spindled cells with scant cytoplasm and oval, elongated, and regular nuclei without nucleoli. The cells are haphazardly embedded in a variably fibro-myxoid stroma, often containing coarse collagen bundles. Mitoses are absent or very rare (<1 mitosis/50HPF). Mast cells are frequently scattered in tumor stroma. In addition, both isolated and classical  NF-associated neurofibromas may contain CD34+/fibroblastic multinucleated giant cells with the cytological features of the so-called “*floret-like cells*”, as commonly seen in spindle cell/pleomorphic lipoma [12]. Immunohistochemically, neurofibroma is typically an S100-protein- and SOX10-positive tumor with a variable expression of CD34 and EMA [1,4,7]. Based on the growth pattern, the following types of neurofibromas can be recognized: (i) localized/nodular neurofibroma; (ii) diffuse neurofibroma; and (iii) plexiform neurofibroma [1,4].

### 3.1. Localized/Nodular Neurofibroma

**Figure 3:**
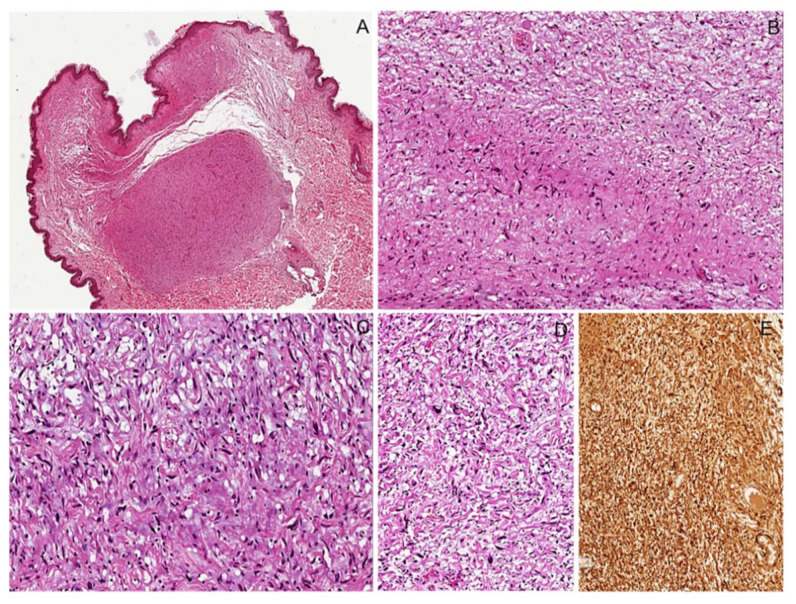
Cutaneous nodular neurofibroma. (**A**) Skin nodule showing a dermal, well-circumscribed, unencapsulated proliferation of spindle cells. (**B**) Neoplastic proliferation showing low to moderate cellularity, in which spindled cells are haphazardly embedded in a fibrillary collagenous stroma. (**C**) Neoplastic cells have hyperchromatic wavy nuclei; wire-like strands of collagen are interspersed among neoplastic cells. (**D**) Mild and focal nuclear atypia can be seen (**E**) Neoplastic cells are diffusely and strongly stained with S100 protein.

**Figure 4:**
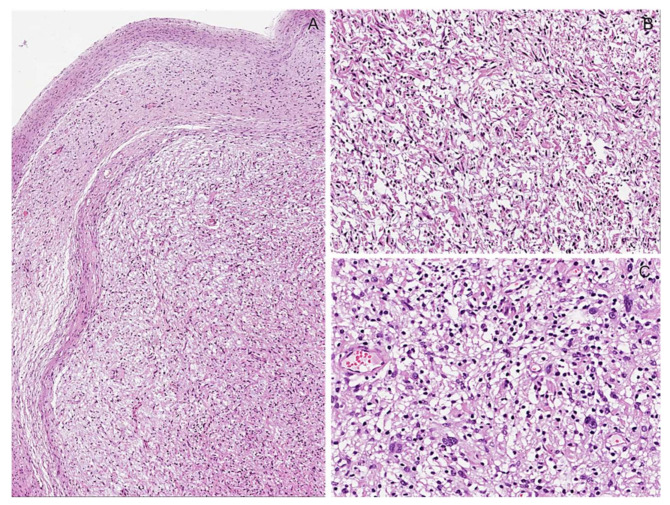
Nodular intraneural neurofibroma. (**A**) Intraneural nodule showing a well-circumscribed, unencapsulated proliferation of spindle cells. (**B**) The neoplastic proliferation is composed of intersecting bundles of spindle cells with hyperchromatic wavy nuclei, sometimes with moderate nuclear atypia (**C**).

This type of neurofibroma is localised in the skin or may be intraneural and, albeit usually isolated, it may be associated to classical NF1 (Table 2 and Table 3). It occurs predominantly in young (20–30 years) to middle-aged adults and usually arises in the skin of the trunk, head/neck region, and extremities (Figure 3). Clinically, it presents as soft, colored papules or a small ovoid/fusiform subcutaneous nodule, usually <2 cm in its greatest diameter, with a glistening tan-white cut section [1]. An anatomic origin from a small-sized nerve of the dermis/subcutaneous tissue cannot be usually documented. It grows slowly as a painless nodule, especially when occurring in the skin, while if it arises from deeply seated nerves, including nerve plexuses and major nerve trunks, it causes sensory or motor deficits related to the affected nerve [1]. Grossly, neurofibroma that arises from major nerves shows a fusiform expansion of the affected nerve and may appear to be encapsulated; conversely, tumors of the small nerves present as well-circumscribed—but not encapsulated—nodules, often lacking an anatomic association with a nerve, and thus they are clinically misdiagnosed as lymph nodes or soft tissue tumors. Histologically, both cutaneous or intraneural neurofibromas present as well-circumscribed and unencapsulated nodules with evidence of a grenz zone if skin-centered, while a peripheral rim of thickened perineurium may be seen in intraneural tumors (Figure 3 and Figure 4). The typical isolated neurofibroma usually shows a low to moderate cellularity and is composed of spindled cells with small wavy or comma-shaped nuclei and poorly defined cellular borders/processes. These cells are haphazardly arranged in a variably fibro-myxoid stroma and may also be arranged, at least focally, in interlacing bundles (Figure 2 and Figure 3). The tumor stroma usually contains collagen fibrils and ropey and/or thicker collagen fibers as well as mast cells and, less frequently, lymphocytes and xanthomatous cells. The incidence of malignant transformation into malignant peripheral nerve sheath tumors is unknown, but it is very low for cutaneous tumors, while intraneural lesions, especially in NF1 patients, may occasionally represent the morphological precursor [1]. Although surgical excision with free margins is usually curative, these lesions can be difficult to be excised in their entirety, and patients should be aware of the possibility of local recurrence.

#### Immunohistochemical Features

Tipically, S-100 protein and SOX10 positivity can be identified in these tumors. CD34 and EMA stain, respectively, the minor components of admixed spindled fibroblasts and perineurial cells, respectively [1].

### 3.2. Diffuse Neurofibroma

**Figure 5:**
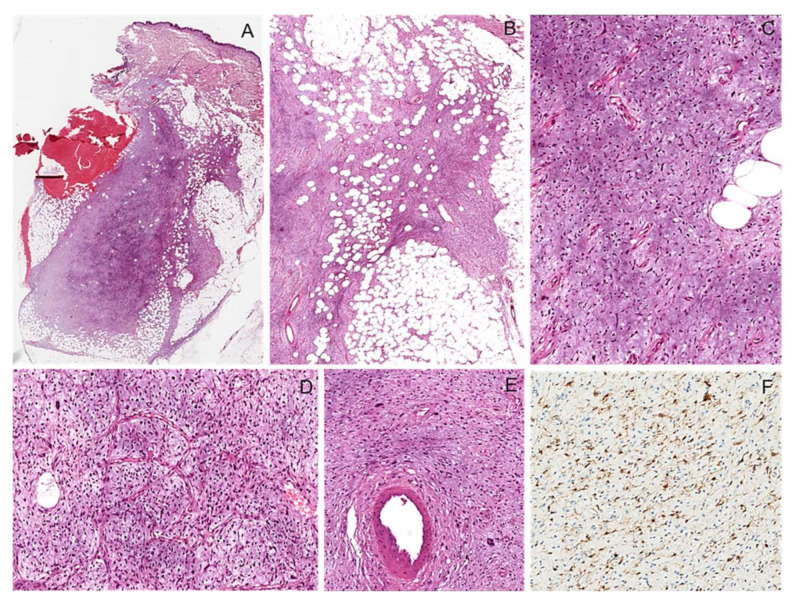
Diffuse neurofibroma. (**A**) Low-magnification image of poorly defined proliferation of spindle cells. (**B**) The neoplastic cells are infiltrating subcutaneous fat. (**C**) The neoplastic cells are embedded in an abundant myxoid stroma. (**D**) A rich plexiform vascular network is seen, mimicking a myxoid liposarcoma. (**E**) Neoplastic cells show focal nuclear atypia. (**F**) Neoplastic cells show cytoplasmic and nuclear staining for S100 protein.

Diffuse neurofibroma occurs more often in the younger age group, including children. Although a number of these lesions may present as isolated/solitary manifestations, they should be regarded as mosaic NF1 lesions or other NF1 stigmata should be carefully searched for [1,9] (Table 2 and Table 4). It usually presents as a plaque-like lesion of the dermis/subcutaneous tissue of the head/neck region. Histologically, diffuse neurofibroma presents as an ill-defined dermal/subcutaneous mass with infiltrative margins entrapping (without destroying) cutaneous adnexal and nerve structures and with extension to the subcutaneous fat, especially along connective tissue septa (Figure 5). The neoplastic cells, closely intermingling with adipocytes, impart the tumor with a honeycomb effect similar to that seen in dermatofibrosarcoma protuberans [1] (Figure 5). In contrast to the other types of neurofibroma, the diffuse type is composed of short spindled to round cells with small, hyperchromatic, wavy or comma-shaped nuclei. The neoplastic cells are haphazardly set in a uniformly fine fibrillary stroma with variably myxoid changes. Some tumors may contain clusters of pseudomeissnerian-body-like structures that seem to be a characteristic feature of diffuse-type neurofibroma [1,7]. Notably, in NF1 patients, it is not uncommon to detect tumor cells admixed with large ectatic vessels and occasionally with branching plexiform-type capillary-like vessels, as seen in myxoid liposarcoma (Figure 5D). In a minority of tumors, neoplastic cells may focally adopt an epithelioid morphology or contain scattered dendritic cells with intracytoplasmic melanin. Although surgical excision with free margins is usually curative, these lesions can be difficult to radically remove. The risk of malignant transformation into a malignant peripheral nerve sheath tumor is very low [13].

#### Immunohistochemical Features

Immunohistochemical analyses reveal the S100-protein and SOX10 positivity of tumor cells.

### 3.3. Plexiform Neurofibroma

**Figure 6:**
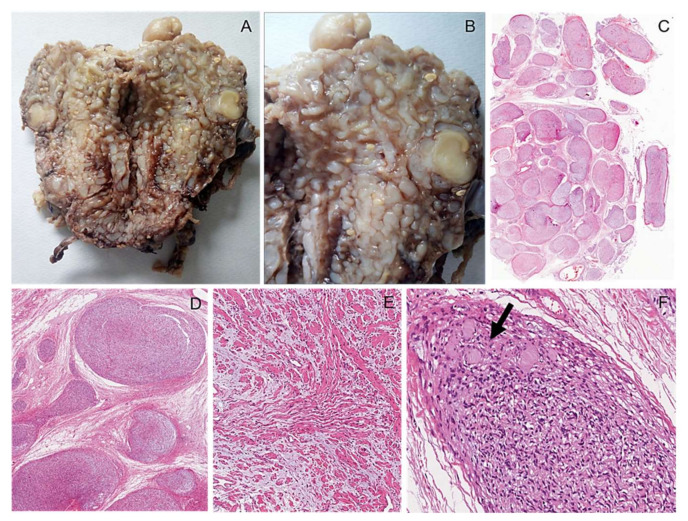
Plexiform neurofibroma. (**A**) Macroscopic appearance of a peritoneal multinodular mass with a plexiform pattern and greyish color; (**B**) the classic “*bag of worms appearance*” can be noticed. (**C**) Low-magnification images showing the typical nodular/plexiform growth pattern. (**D**) High-magnification image showing serpentine nerve-like structures embedded in an abundant myxo-edematous stroma rich in haphazardly arranged thick collagen fibers (*shredded carrots appearance*) (**E**). Occasional Wagner–Meissner corpuscles (lamellar growth pattern) can be observed (arrow) (**F**).

Plexiform neurofibroma is a type of neurofibroma arising virtually only in NF1 patients (up to 40% of all patients with NF1), and thus its histologically proven diagnosis is considered pathognomonic of this syndrome [1] (Table 5); notably, even an isolated/solitary plexiform neurofibfroma (i.e., a plexiform neurofibroma truly presenting without other NF1 stigmata after careful clinical, laboratory and instrumental work-up, should be regarded as a manifestation of mosaic NF1 (Table 2); occasionally superficial and deep soft tissues of an entire extremity are involved, giving rise to the rare condition known as “*elephantiasis neuromatosa*”, which is characterized by hyperpigmented, pendulous folds of the overlying skin and is often associated with bone hypertrophy [1]. Grossly, plexiform neurofibroma appears as a multinodular mass composed of irregular/serpentine structures reminiscent of intermingling nerve fascicles (*bag of worms* appearance) (Figure 6). Histologically, the tumor consists of multinodular and serpentine nerve-like structures with abundant myxo-edematous stroma containing thick haphazardly arranged collagen fibers (*shredded carrots* appearance) [1]. Notably, tumors cells are usually associated with thicker collagen bundles, often simulating bundles of smooth muscle cells. Interestingly, the neoplastic proliferation may extend beyond the nerve-like structures into the surrounding tissues with a morphology closely resembling diffuse-type neurofibroma; this latter growth pattern may be occasionally predominant, obscuring the underlying plexiform architecture. The importance of a correct diagnosis of plexiform neurofibroma relies not only on its association with NF1 but also in its highest risk of malignant transformation among all types of neurofibromas [1].

### 3.4. Potential Morphological Pitfalls of Malignancy in Neurofibromas: Cytological Atypia and Hypercellularity

**Figure 7:**
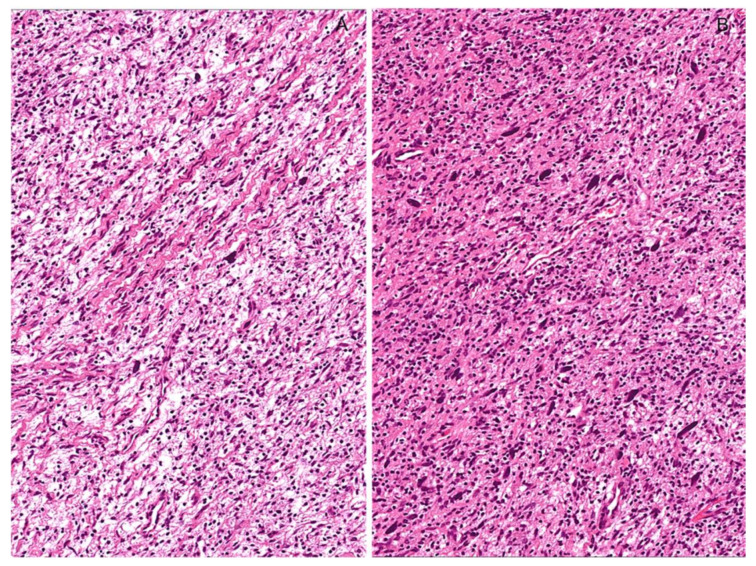
Neurofibroma with cytological atypia. Low (**A**) and higher (**B**) magnifications showing diffuse nuclear atypia; hypercellularity, mitoses, and necrosis are not observed.

Despite the different types, both sporadic and NF1-associated neurofibromas may contain focal or diffuse nuclear atypia in the absence of increased mitotic activity (≥1 mitosis/50 HPF) and/or hypercellularity. Although alarming, cytological atypia alone, in the context of an otherwise specified neurofibroma, is not by itself a criterion of malignancy [1,6]. Nuclear atypia consists of nuclear enlargement (2–3-fold or more), hyperchromasia, and irregular chromatin distribution (Figure 7); multinucleated/bizarre cells are in the spectrum of cytological atypia. Although similar to the so-called “*degenerative atypia*” seen in ancient schwannoma, some authors favor the use of the term “*neurofibroma with cytological atypia*” rather than “*ancient neurofibroma*”, especially in NF1 patients [1]. This is mainly due to the fact that it is impossible to morphologically distinguish “*degenerative atypia*” from the “*malignancy-related atypia*” that is one the morphological criteria for malignant transformation along with an increased number of mitoses, hypercellularity, and a loss of neurofibroma architecture. Accordingly, we recommend that all neurofibromas showing cytological atypia alone should be extensively sampled to rule out the presence of areas with hypercellularity, increased mitotic activity, and/or tumor necrosis. Potential diagnostic pitfalls may arise when inflammatory cells, especially lymphocytes and histiocytes, may be extensively found among neoplastic cells, imparting a neurofibroma with a false hypercellular appearance. In addition, it should be emphasized that the diagnosis of neurofibroma with cytological atypia should be rendered with caution when a pathologist is dealing with small biopsies from NF1 patients. Based on these considerations, radiological correlation is crucial, suggesting obtaining multiple core biopsies from areas with the suspicion of malignant transformation to avoid the risk of tumor undersampling. As neurofibromas with cytological atypia are treated conservatively, even in presence of positive margins [1], some authors discourage the term “*atypical neurofibroma*” to avoid confusion among clinicians who could consider it as a premalignant lesion [1].

**Figure 8:**
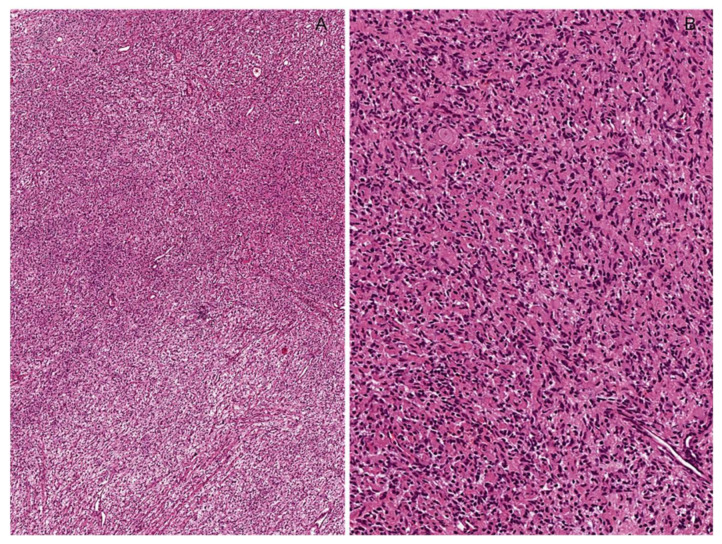
Cellular Neurofibroma. (**A**) Low-magnification image of a hypercellular tumor with a fascicular arrangement of the neoplastic cells. (**B**) The absence of cytological atypia and mitoses is against the diagnosis of malignancy.

Apart from cytological atypia, the term “*cellular neurofibroma*” should be restricted to those tumors in which hypercellularity is the only worrisome feature and in the absence of increased mitotic activity, cytological atypia, and/or the loss of neurofibroma architecture [6]. Histologically, cellular neurofibroma can be appreciated at low magnification for the presence of areas with greater cellularity and the fascicular arrangement of neoplastic cells [1] (Figure 8). Similar to cytological atypia, hypercellularity alone is not by itself a criterion of malignancy. Cellular neurofibroma should be extensively sampled to rule out the presence of areas with cytological atypia and/or increased mitotic activity. As in neurofibroma with cytological atypia, it should be emphasized that the diagnosis of cellular neurofibroma should be rendered with caution when a pathologist is dealing with small biopsies from NF1 patients. Similarly, radiological correlation is crucial, suggesting obtaining multiple core biopsies from those areas with a high suspicion of malignant overgrowth to avoid the risk of tumor undersampling. Cellular neurofibroma is treated conservatively, even in presence of positive margins.

#### Immunohistochemical Features

Although it is not routinely recommended, immunohistochemistry may be reassuring, revealing a low percentage of Ki67-positive cells and p53 positivity restricted to a few cells [6].

## 4. Atypical Neurofibromatous Neoplasm with Uncertain Biologic Potential (ANNUBP)

**Figure 9:**
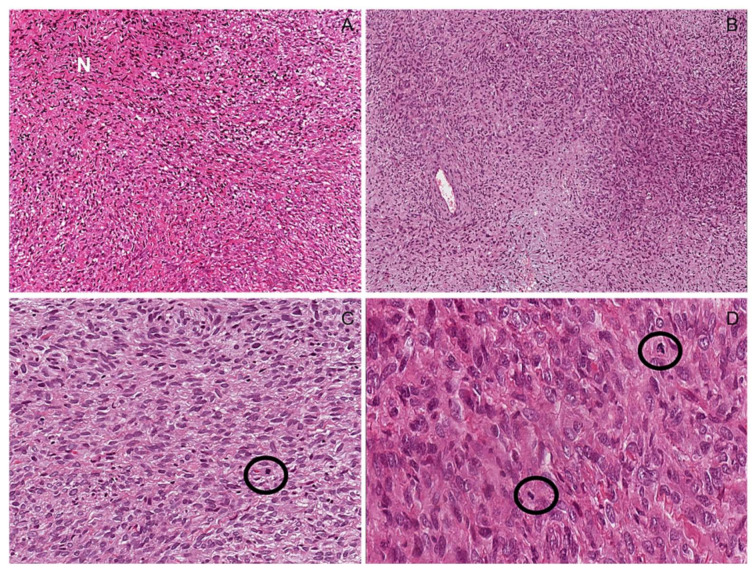
Atypical neurofibromatous tumor with uncertain biological behavior. (**A**) Low-magnification image showing hypercellular tumor with focal area reminiscent of neurofibroma (N). (**B**) Tumor area with alternating hypercellular and hypocellular areas. (**C**,**D**) Mitoses (circles) and moderate nuclear atypia are seen (**D**); tumor necrosis was absent.

The term “*atypical neurofibromatous neoplasm with uncertain biologic potential (ANNUBP)*” has been recently coined [6] for a subset of NF1-associated neurofibromatous tumors that exhibit at least two of the following features: (i) nuclear atypia; (ii) hypercellularity; (iii) increased mitotic activity (>1 mitosis/50 HPF but <3 mitoses/10 HPF); and (iv) a variable loss of neurofibroma architecture (i.e., the presence of herringbone and/or fascicular storiform growth patterns) (Figure 9) (Table 6). These tumors are a provisional diagnostic category created on the evidence of their virtually absent (or very low) risk of metastasis. Similar to neurofibromas with cytological atypia or hypercellularity alone, the diagnosis of ANNUBP should be rendered with caution on small biopsies, suggesting to clinicians that the examined tumor does not fit all the morphological criteria of malignancy. If the radiological suspicion of malignancy is high, a close clinical follow-up and/or additional bioptic sampling is necessary.

### Immunohistochemical Features

Although immunohistochemical analyses are not helpful for the diagnosis of ANNUBP, a variable to complete loss of S100 protein/SOX10 expression and a loss of the CD34-positive fibroblastic network, typically found in all types of neurofibromas, may be suggestive [6].

## 5. Malignant Transformation in Neurofibromas

Although the risk of transformation into a malignant peripheral nerve sheath tumor (MPNST) is very low (if any) in isolated/solitary cutaneous/nodular or diffuse neurofibromas, it is well-documented in NF1-associated intraneural and/or plexiform neurofibromas [1]. The lifetime risk for the development of MPNST in NF1 patients has been calculated as 8–16% in two population-based studies [14,15,16]. Most NF1 patients develop MPNST during their 3rd to 4th decades, even if malignant transformation can also occur in childhood [notably, children/adults harbouring NF1 gene deletions (i.e., the so-called “NF1 microdeletion syndrome” (Table 2), are at higher risk of malignant transformation]. In the majority of cases, the diagnosis of malignant transformation is straightforward in that it is represented by a high-grade sarcoma, often arising abruptly from areas of classic neurofibroma into hypercellular areas with diffuse cytological atypia, increased mitotic activity (often >10 mitoses/10 HPF), and/or tumor necrosis. Less commonly, the transition is more challenging, being represented by areas of well-differentiated neoplasms with the morphological features of “*atypical neurofibromatous neoplasms with uncertain biologic potential* (ANNUBP)” in which, however, the mitotic activity ranges from 3 to 9 mitoses/10 HPF in the absence of tumor necrosis (low-grade MPNST). In a minority of cases, all the morphological spectrum ranging from a classic neurofibroma to ANNUBP, to low-grade and high-grade MPNST, can be seen.

## 6. Schwannoma

**Figure 10:**
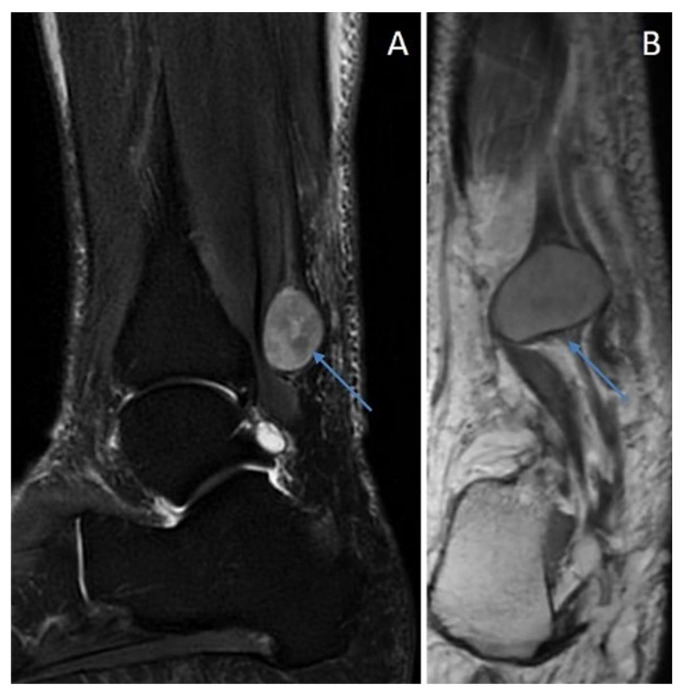
Solitary schwannoma: round mass (arrows) of the posterior tibial nerve. (**A**) DP fat sat sagittal view and (**B**) T1 post-enhanced sagittal view.

**Figure 11:**
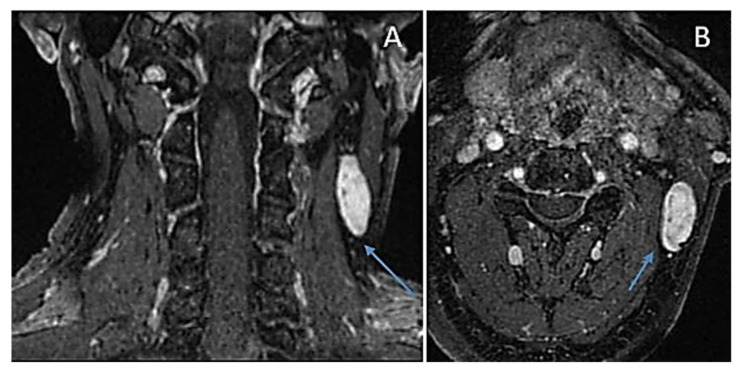
Solitary schwannoma of accessory spinal nerve. LAVA post-enhanced MRI: coronal view (**A**) and axial view (**B**) showing a non-homogenous well-defined mass (arrows) strongly enhancing along the tract of the accessory spinal nerve.

**Figure 12:**
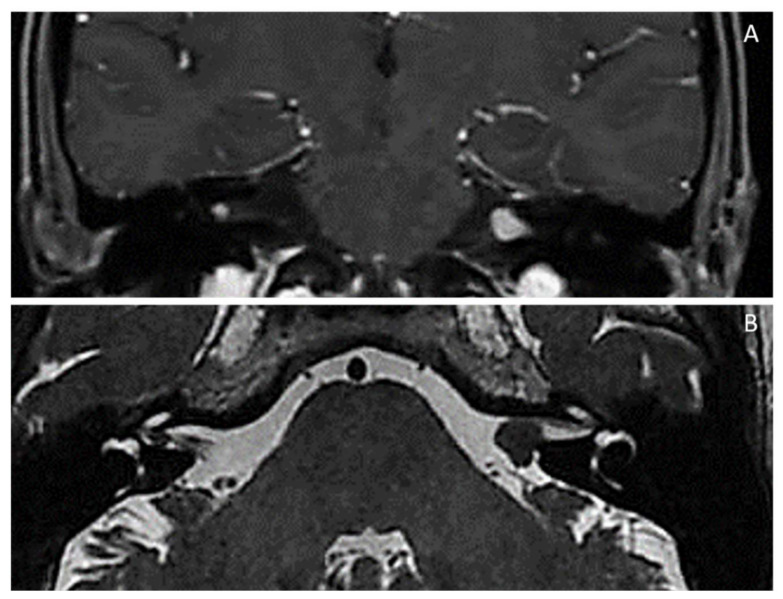
Patient with NF2-related schwannomatosis (ex-NF2): (**A**,**B**) bilateral acoustic schwannoma is pathognomonic.

Schwannoma is an encapsulated benign nerve sheath tumor arising from small or large nerves that is composed almost exclusively of spindled cells showing the morphological, immunohistochemical, and ultrastructural features of Schwann cells [1,4]. Although most cases (90% of cases) arise as isolated/solitary lesions (Figure 10 and Figure 11), their occurrence within the context of NF2-related-schwannomatosis or NF2/MERLIN schwannoma predisposing syndrome (formerly, NF2), SMARCB1/LZTR1-related-schwanomatoses or SMARCB1/LZTR1 schwannoma predisposing syndromes (formerly, schwannomatosis) and 22q-related-schwannomatosis or 22q schwannoma predisposing syndrome (Table 7) is well-known [1,8]. Recently, the umbrella term “*schwannomatosis”* has been proposed to encompass the spectrum of syndromes characterized by the development of (or the predisposition to develop) schwannomas, [as opposed to the spectrum of syndromes characterised by the development of (or predisposition to develop) neurofibromas: i.e., NF1 and related disorders], including NF2 (now called NF2-related-schwannomatosis or NF2/MERLIN schwannoma predisposing syndrome) and schwannomatosis (now called SMARCB1-related and LZTR1-related-schwannomatoses or SMARCB1/LZTR1 schwannoma predisposing syndromes) (Table 7) [3]. The new classification of schwannomatoses is based on the coupling of the causative genes (i.e., *NF2, SMARCB1* and *LZTR1*) and on the histological hallmark of these syndromes (i.e., schwannomas) [3]. Accordingly, the term “*neurofibromatosis type 2 (NF2)*” has been replaced by “*NF2*-related schwannomatosis”, while the so-called “schwannomatoses” (SWNTNs) have been reclassified as: (i) “SMARCB1-related-schwannomatosis (SMARCB1 schwannoma predisposing syndrome); (ii) “LZTR1-related-schwanomatosis” (LZTR1 schwannoma predisposing syndrome); and (iii) 22q-related-schwannomatosis (22q schwannoma predisposing syndrone); further categories refelect the incomplete knowledge of the whole spectrum of disorers by creating a 4th group—“schwannomatosis-not-otherwise-specified” or “schwannoma predisposing syndromes not otherwise specified” (NOS); and a 5th group including (v) “schwannomatosis-not-elsewhere-classified” or “schwannoma predisposing syndromes not elsewhere classified” (NEC) (Table 2). From a pathological point of view, this new classification implies that clinicians may ask surgical pathologists to perform molecular genetic tests on schwannoma tissues from patients suspected of having any type of schwannomatosis [3]. With the exception of bilateral vestibular schwannomas (pathognomonic of “*NF2*-related schwannomatosis”) (Figure 12) (Table 8), the isolated/solitary schwannoma, as it occurs with its counterpart within the spectrum of NF1-related disorders (i.e., the neurofibroma), should/could be regarded as a manifestation of mosaic NF2/SMARCB1/LZTR1 or 22q-related-schwannomatoses (or schwannoma predisposing syndromes) and thus molecular genetic testing should be performed on the referred specimen(s) (Table 7).

Schwannomas usually present as slowly growing painless masses, but they may be painful if associated with large-sized nerves [1]. Grossly, schwannomas present as well-circumscribed, small-sized (often <5 cm) masses surrounded by a capsule consisting of epineurium. Their gross appearance usually depends on the size of the affected nerve. In case of small nerves, schwannomas present as fusiform-shaped masses, often obscuring the pre-existing nerve and thus mimicking the overall appearance of neurofibromas [1]; it is not uncommon that these schwannomas are pre-operatively misdiagnosed as lymph nodes. Conversely, if the affected nerve is large in size, schwannomas form eccentric masses that stretch the fibers of the nerve of origin [1]. On cut section, they are typically pinkish-white or yellowish in color and often show degenerative changes, such as foci of hemorrhage and cystic degeneration. Retroperitoneal and mediastinal schwannomas tend to form larger masses with microcystic changes, calcifications, fibrosis, and hemorrhages. Based on histological features and architectural growth pattern [1], the following types of schwannoma are recognized [1]: (i) classic schwannoma; (ii) schwannoma with degenerative/ancient changes (“*ancient schwannoma*”); (iii) cellular schwannoma; (iv) plexiform schwannoma; (v) epithelioid cell schwannoma; and (vi) reticular/microcystic schwannoma.

### 6.1. Classic Schwannoma

**Figure 13:**
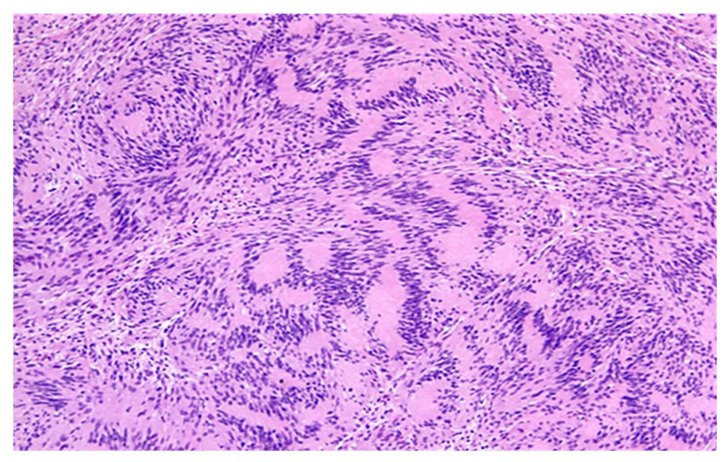
Classic schwannoma (Antoni A area). The diagnostic clue is the presence of numerous Verocay bodies.

Classic schwannoma is by far the most common type among all schwannomas and may occur as an isolated/solitary lesion (think always about mosaicism for the schwannomatoses genes) or within the context of any type of syndromic schwannomatoses (Table 8). The histological hallmark of classic schwannoma is the presence of alternating hypercellular (so-called “*Antoni A areas*”) and hypocellular (so-called “*Antoni B areas*”) areas, variably represented in the same tumor; the former areas may blend imperceptibly or abruptly into the latter ones [1]. Antoni A areas are composed of compact spindled cells with wavy nuclei, dense chromatin, and indistinct cytoplasm, often arranged in short and intersecting fascicles, sometimes forming meningioma-like whorls [1]. Notably, in these areas the neoplastic cells characteristically exhibit nuclear palisading around anucleated central spaces filled with eosinophilic fibrillary cytoplasmic processes (the so-called “*Verocay bodies*”) [1,4] (Figure 13). Conversely, Antoni B areas are less cellular and are composed of spindled to ovoid-shaped cells, haphazardly set in a loose myxoid stroma with microcystic changes, inflammatory cells (lymphocytes and histiocytes), and collagen fibers; in these hypocellular areas, there are numerous large-sized and irregularly spaced blood vessels with thickened hyalinized walls, often filled with fibrin. Mitoses are usually absent or rare. Interestingly, schwannomas arising in the gastrointestinal tract typically show a lymphocytic rim at their periphery.

#### Immunohistochemical Features

Immunohistochemically, unlike neurofibroma, schwannoma exhibits a diffuse and strong S100 protein expression in that it is almost exclusively composed of spindle cells with schwannian differentiation [1,4,6,17]. Although S100 protein expression is typically weaker and more patchy in Antoni B areas, it may be helpful when dealing with a schwannoma exhibiting extensive degenerative changes, in which the amount of fibro-myxoid stroma and/or cystic stromal degeneration can make diagnosis more challenging. SOX-10 is an additional marker of schwannoma that is expressed in the majority of cases [1]. Interestingly, occasional aberrant expressions of cytokeratins, desmin, and TTF-1 can be found [18,19]. Notably, a mosaic expression pattern of INI1 has been found to be helpful in distinguishing schwannomas, in the context of the different types of schwannomatosis, from apparently sporadically occurring and solitary tumors as [20].

### 6.2. Schwannoma with Degenerative/Ancient Changes (“Ancient Schwannoma”)

**Figure 14:**
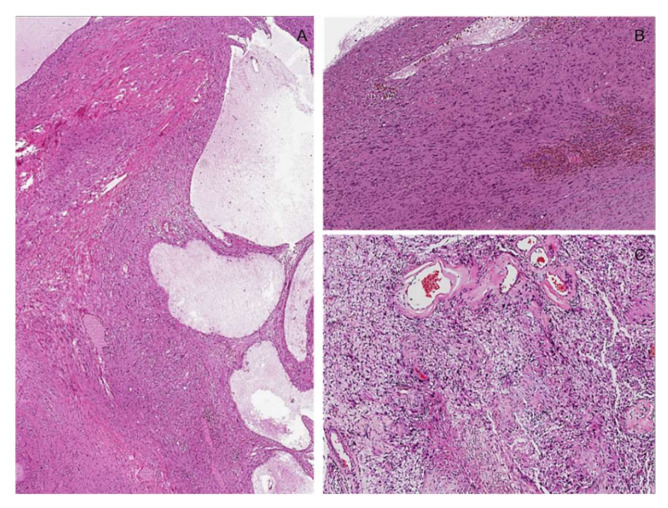
Schwannoma with “*ancient*” changes. (**A**) Low-magnification image showing cystic stromal changes and stromal hyalinization. (**B**) Hemorrhages can be observed intermingling with tumor cells that exhibit nuclear atypia. (**C**) Thick-walled vessels are seen.

This term should be reserved to those schwannomas that histologically show degenerative-type atypia as well as marked stromal changes (Table 9). These tumors often are large-sized and long-standing and usually affect the deep soft tissues, especially the retroperitoneum or head and neck region [1,21,22]. Histologically, their characteristic features are nuclear atypia of the “degenerative type” as well as other degenerative changes, including cystic stromal changes, hemorrhages, calcifications, and diffuse stromal hyalinization [1] (Figure 14). Numerous histiocytes/siderophages are often encountered, intermingling with tumor cells. The striking nuclear atypia is the most alarming feature of ancient schwannoma, representing a diagnostic pitfall of malignancy; tumor cells usually contain large, hyperchromatic, and multilobulated nuclei, but they characteristically lack mitotic activity. The disproportion between nuclear atypia and mitotic figures is a diagnostic clue of benignancy [1].

### 6.3. Cellular Schwannoma

**Figure 15:**
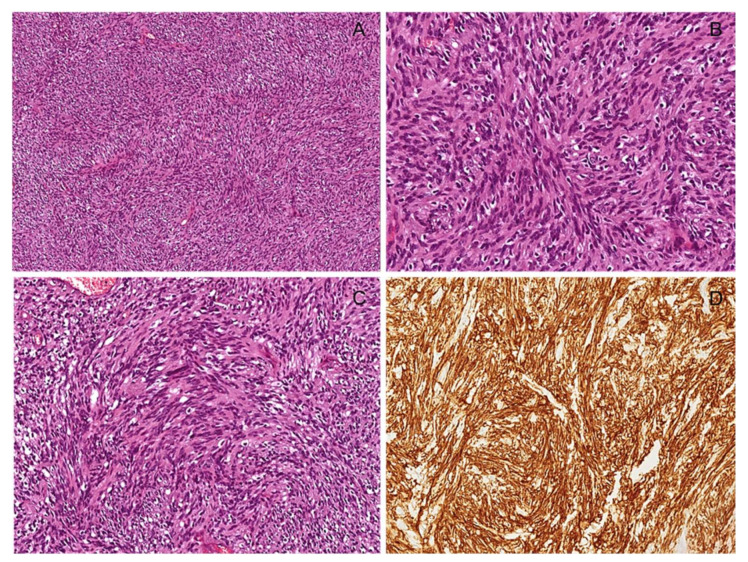
Cellular schwannoma. (**A**) Low-magnification image showing a hypercellular tumor composed uniformly of Antoni A areas and lacking hypocellular Antoni B areas. (**B**) Tumor is usually composed of bland-looking spindle cells arranged in short intersecting fascicles. (**C**) As an additional characteristic feature, long sweeping fascicles of spindle cells arranged in a storiform growth pattern may be seen. (**D**) Unlike other spindle cell tumors, tumor cells are diffusely and strongly stained with S100 protein.

Schwannomas that are exclusively or predominantly composed of Antoni A areas and lacking Verocay bodies (or only focally seen) are labeled as “*cellular schwannomas*” (Table 10) [1,4]. Unlike the classic type, cellular schwannoma more frequently affects the deep soft tissues, including the retroperitoneal and posterior mediastinal regions, with only a minority of cases occurring at the extremities [23]. The macroscopic appearance is similar to that of classic-type schwannoma, but occasionally a multinodular or plexiform architecture can be appreciated. Histologically, it is composed uniformly of hypercellular Antoni A areas, albeit a minority of tumors can contain Antoni B areas accounting for less than 10% of the entire tumor (Figure 15). Like in classic-type schwannoma, Antoni A areas are composed of short intersecting fascicles; however, an additional characteristic feature is the presence of long sweeping fascicles arranged in a herringbone and/or storiform growth pattern, closely mimicking fibrosarcoma or leiomyosarcoma [1]. Malignancy can also be seriously considered because mitotic activity is higher (usually <4–5 mitoses/10 HPFs) than that seen in classic-type schwannoma, and foci of necrosis can be found in up to 10% of cases [1,4]. Another alarming feature is the presence of infiltrative margins that can produce bone erosion [1]. Due to the presence of the above-mentioned “alarming” features, cellular schwannoma can be misdiagnosed as a spindle cell sarcoma, especially if the pathologists are unfamiliar with soft tissue tumors. The benign nature of the tumor is supported by the following features: (i) sharply demarcated margins (often encapsulation); (ii) a focal presence of Antoni B areas; (iii) disproportion between hypercellularity, nuclear pleomorphism, and the amount of necrosis and mitoses; and (iv) strong and diffuse S100 protein and SOX10 immunoexpression. The cellular schwannoma has a higher rate (4–40%) of local recurrence, likely due to its deeper anatomic location that makes a complete surgical excision difficult [1].

#### Immunohistochemical Features

Immunohistochemistry is crucial for achieving a correct diagnosis, especially on small biopsies, as virtually no bland-looking spindle cell sarcoma is diffusely and strongly stained with S100 protein and/or SOX10; typically, the nuclear expression of H3K27me3 is retained, and the lack of expression of epithelial and myogenic markers as well as CD117/DOG1 is useful in the differential diagnosis with other tumor entities, such as synovial sarcoma, leiomyosarcoma, and GIST, respectively.

### 6.4. Plexiform Schwannoma

**Figure 16:**
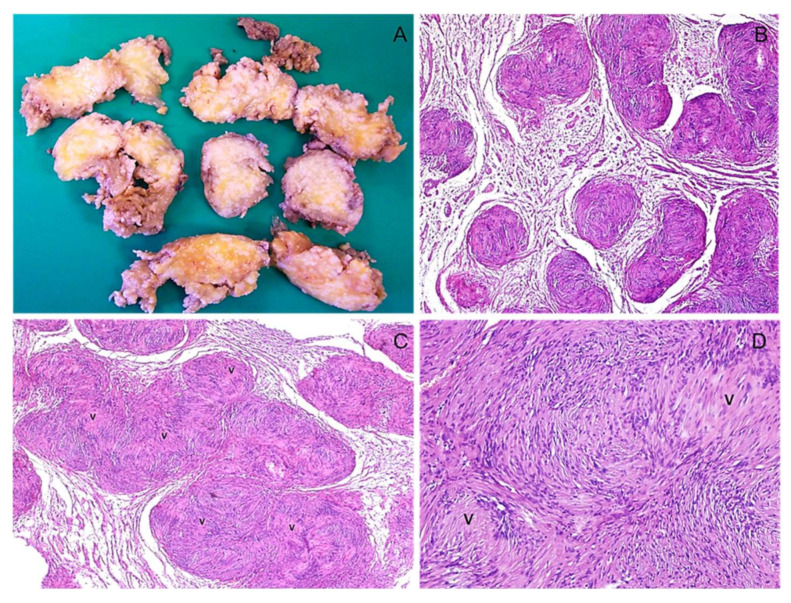
Plexiform schwannoma in a patient with NF2-related schwannomatosis. (**A**) Gross appearance of an abdominal plexiform schwannoma showing a multinodular mass with a plexiform pattern and greyish color. (**B**) At low magnification, the plexiform growth pattern can be appreciated. Verocay bodies can be observed at both low (**C**) and higher (**D**) magnifications.

Plexiform schwannomas represent an uncommon variant of schwannoma (about 5% of all cases) that characteristically exhibit a multinodular/plexiform architecture, often appreciated at macroscopic examination (Table 11). Unlike plexiform neurofibromas, which most often arise within the context of classical NF1 and are virtually pathognomonic to NF1, most plexiform schwannomas are (at least initially) isolated/solitary, with only a few cases arising within the context of NF2-related schwannomatosis (NF2/MERLIN schwannoma predisposing syndrome) [1,4,9,24,25,26,27,28,29]; one must think, however, in the cases of truly isolated/solitary lesions (after extensive work-up), about mosaicism for the schwannomatoses genes (Table 7). They commonly affect the skin (dermis/subcutis) of the head and neck region and the distal extremities; more rarely, deep soft tissues are involved. Although usually encapsulated, they may lack the capsule. Histologically, the hallmark is the multinodular/plexiform growth pattern. Apart from this peculiar growth pattern, plexiform schwannoma is usually a cellular schwannoma, being mainly composed of Antoni A areas (Figure 16) [24,25,26,27,28,29]. Pathologists should be aware of this hypercellularity to avoid a misdiagnosis of sarcomatous transformation/overgrowth in a plexiform schwannoma. The rare occurrence of plexiform schwannomas in deeper soft tissues or in large peripheral nerves may represent a further diagnostic challenge, as they may exhibit increased cellularity and mitotic count and thus pose differential diagnostic problems with malignant peripheral nerve sheath tumors [24,25,26,27,28,29]. The absence of tumor necrosis, significant nuclear atypia, and the relatively low number of mitoses are in contrast with malignancy [1]. Although a higher rate of local recurrence has been reported, additional studies on larger series are needed.

#### Immunohistochemical Features

The strong and diffuse expression of S100 protein and SOX10, along with the retained expression of H3K27me3 in tumor cells, are helpful to exclude a malignant peripheral nerve sheath tumor [1].

### 6.5. Epithelioid Cell Schwannoma

**Figure 17:**
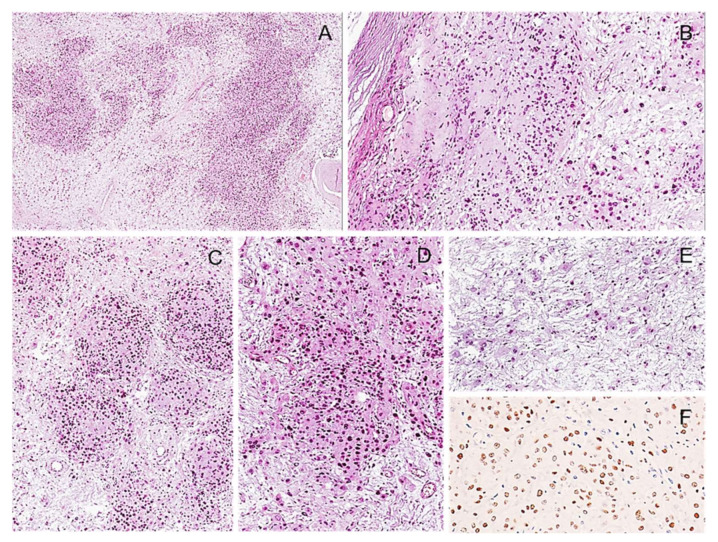
(**A**) Low-magnification image showing a moderately cellular lesion composed of epithelioid cells set in a myxoid stroma. (**B**) At higher magnification, small- to intermediate-sized rounded to epithelioid cells with rounded hyperchromatic nuclei and abundant pale/eosinophilic cytoplasm and well-defined cellular borders are seen. (**C**) Neoplastic cells are arranged in small nests and cords. (**D**) Tumor cells may occasionally exhibit nuclear pleomorphism. (**E**) Epithelioid cell schwannoma may contain a variable amount of large-sized deciduoid-like cells. (**F**) Diffuse immunoreactivity for SOX10 is shown.

Epithelioid cell schwannoma is a rare variant of schwannoma composed exclusively/predominantly of epithelioid tumor cells showing schwannian differentiation (Table 12) [1]. This tumor arises as an isolated/solitary lesion, usually sporadically, without any evident association with any of the different forms of schwannomatoses [1,4]. Epithelioid schwannoma is usually a well-circumscribed and small-sized tumor arising in the skin or in the superficial soft tissues, especially of the extremities. Histologically, it is composed of small- to intermediate-sized rounded to epithelioid cells with abundant eosinophilic cytoplasm and well-defined cellular borders; nuclei are rounded and contain prominent nucleoli and nuclear pseudoinclusions; and a variable number of large-sized cells with the morphology of deciduoid-like cells can be seen (Figure 17) [30,31,32]. The neoplastic cells, often set in a fibro-myxoid stroma, are usually arranged singly or in small nests or cords. A characteristic feature is the presence of collagen rosettes consisting of the condensation of neoplastic cells around central cores of dense collagen. These rosettes can be occasionally seen in classic-type schwannoma (the so-called “neuroblastoma-like schwannoma) [33,34,35]. The diagnosis of epithelioid cell schwannoma can be suggested by the identification, at least focally, of tumor areas with the morphology of classic schwannoma. Nuclear atypia and mitoses can be documented, but they do not represent signs of malignancy. Actually, epithelioid cell schwannoma is considered a benign tumor with a very low risk of malignant transformation, similar to that documented in the other types of schwannomas.

#### Immunohistochemical Features

As for the other types of schwannoma, even epithelioid cell schwannoma is strongly and diffusely stained with S100 protein and SOX10 [1]. Notably, in the last years there is convincing evidence that approximately 40% of cases lack nuclear expression of SMARCB1/INI1 [36].

### 6.6. Unusual Features in Schwannomas

Schwannomas may rarely contain glands and epithelial structures that are believed to represent “true” epithelial differentiation rather than entrapped normal elements [37]. In addition, large cysts lined by Schwann cells with rounded/epithelioid morphology mimicking epithelial differentiation may be occasionally encountered (pseudoglandular schwannomas) [38]. Occasionally, schwannomas may exhibit, at least focally, a small cell component with scant cytoplasm arranged around collagen-filled spaces or around vessels, forming collagen rosettes and/or perivascular collagen pseudorosettes (neuroblastoma-like schwannoma) [39,40]. Like in neurofibromas [41], rare cases of schwannomas exhibiting intralesional mature adipocytes, along with lipoblast-like cells with signet-ring cell morphology, have been labeled as “*schwannomas with lipoblastic differentiation*” [42].

### 6.7. Malignant Transformation in Schwannoma

Schwannomas are benign tumors with only a few cases exhibiting well-documented malignant transformation [43,44,45,46,47,48]. Unlike in neurofibromas, malignancy in schwannomas is mainly represented by malignant peripheral nerve sheath tumors with epithelioid cell morphology [47]. All these malignant cases seem to arise sporadically, without evidence of any type of schwannomatosis [1].

## 7. Perineuriomas

Perineurioma is a benign peripheral nerve sheath tumor composed almost entirely of neoplastic cells that exhibit morphological, immunohistochemical, and ultrastructural features consistent with perineurial differentiation [1,4]. Different from other peripheral nerve sheath tumors, perineurioma is a sporadic tumor with only a few cases reported to be associated with NF1 or other types of schwannomatosis [1]. Based on its location, perineurioma can be distinguished as: (i) intraneural perineurioma or (ii) soft tissue perineurioma [49,50].

### 7.1. Intraneural Perineurioma

Intraneural perineurioma, also known as “localized hypertrophic neuropathy”, is a rare benign tumor growing within and expanding the nerve fascicles (Table 13) [49]. This tumor primarily affects the extremities of young adults and children, and it usually presents as a solitary, slowly growing, painless mass, often causing neurologic motor and/or sensory defects. The most commonly affected nerves are the ulnar, median, peroneal, sciatic, and radial nerves [1,49]. Grossly, the involved nerve shows segmental and fusiform expansion with variable extension; if the affected nerve is small in size, a plexiform-type architecture can be appreciated. The histological hallmark is a proliferation of bland-looking spindle-shaped cells with wavy to round nuclei arranged in concentric layers (onion-bulb-like arrangement) surrounding the more centrally located Schwann cells and axons [1,4]. This growth pattern is better appreciated when evaluating cross, rather than longitudinal, sections of the involved nerve. The clinical behavior is benign.

#### 7.1.1. Immunohistochemical Features

Immunohistochemically, perineurial cells are stained with EMA; immunoreactivity has also been reported with claudin-1 and GLUT-1 [1].

#### 7.1.2. Molecular Features

Although intraneural perineurioma had been considered as a reactive process (localized hypertrophic neuropathy), the identification of clonal cytogenetic abnormalities involving chromosome 22 as well as the recent discovery of *TRAF7* mutations in 60% of cases seems to confirm its neoplastic nature [51,52,53,54,55].

### 7.2. Soft Tissue (Extraneural) Perineurioma

**Figure 18:**
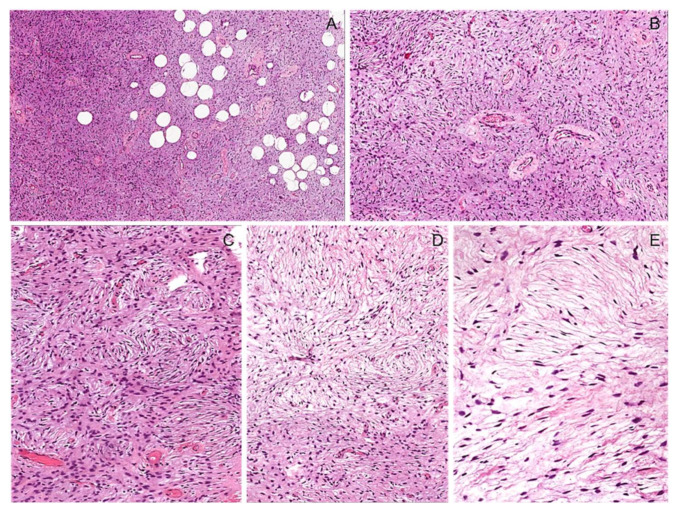
Soft tissue perineurioma. (**A**) Low-magnification image showing a moderately cellular bland-looking spindle cell tumor with focal infiltrative margins into the subcutaneous adipose tissue. (**B**) Perivascular hyalinization is seen. (**C**) Bland-looking spindle cells arranged in a storiform/whorled or reticular (**D**) growth pattern and set in a fibro-myxoid stroma. (**E**) High-magnification image showing bland-looking slender fibroblast-like cells with long bipolar cytoplasmic processes.

**Figure 19:**
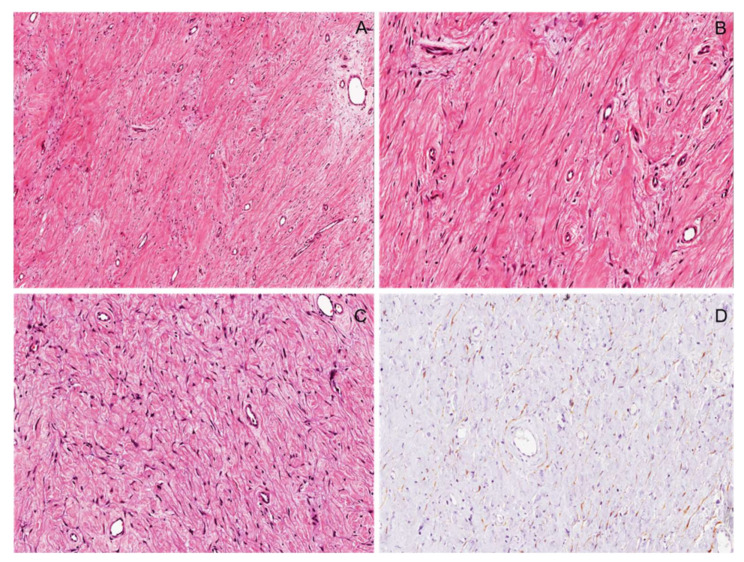
Sclerosing perineurioma. (**A**) Low-magnification image showing a hypocellular tumor composed of spindles set in a fibrosclerotic stroma. (**B**) Tumor cells are arranged in fascicular and/or reticular (**C**) growth patterns. (**D**) Neoplastic cells showing diffuse immunoreactivity for EMA.

Soft tissue perineurioma presents as a well-circumscribed mass, usually occurring in the subcutaneous tissue of the extremities and trunk; less commonly, it is located in the dermis, deep soft tissue, or in visceral locations (Table 14) [1,50]. This tumor usually occurs in middle-aged adults and is slightly more frequent in females; children are rarely affected. The clinical presentation is that of a painless and slowly growing mass. Grossly, soft tissue perineurioma is a well-circumscribed unencapsulated mass ranging in size from 1 cm to 20 cm. The cut surface shows a firm or rubbery consistency and a yellow-tan to whitish color. Histologically, it is a well-circumscribed (rarely infiltrative) and unencapsulated tumor composed of a proliferation of slender fibroblast-like cells with long bipolar cytoplasmic processes variably arranged into a fascicular, storiform, whorled, or lamellar (Pacinian) growth pattern (Figure 18) [1,50]. Cellularity is variable, ranging from paucicellular to densely cellular areas. The tumor stroma is usually collagenized, with focal edematous or myxoid changes. Nuclear atypia, mitoses, and tumor cell necrosis are virtually absent; however, larger tumors may exhibit ischemic-type foci of necrosis. Rare cases may exhibit a minor component of plumper and even epithelioid tumor cells [56]. An intratumoral lipomatous component, including an unusual “pseudolipoblastic” morphology mimicking liposarcoma, has also been reported [57]. Occasionally, some cases exhibiting ossification foci, granular cell change, and Pacinian-like bodies have been described [58,59]. Apart from the classic type, soft tissue perineurioma may exhibit in at least two different histological variants, reticular and sclerosing perineurioma [60,61,62,63]. The former is characteristically composed of slender spindled cells, usually set in a myxo-edematous stroma showing cytoplasmic anastomosing processes, resulting in a reticular or lace-like appearance [60,61]. Sclerosing perineurioma is an unusual variant, mainly occurring in young male adults and involving almost exclusively the superficial soft tissues of the hand. Histologically, it is composed of a proliferation of spindled to rounded/epithelioid cells with pale cytoplasm, indistinct cell borders, and slightly hyperchromatic nuclei arranged in corded, trabecular, or whorled growth patterns and set in an abundant fibro-sclerotic stroma (Figure 19) [62,63]. Despite the morphological variant, perineurioma may exhibit potential alarming/atypical features in approximately 10–20% of cases, including degenerative nuclear atypia (pleomorphic and multinucleated cells with nuclear pseudoinclusions), mitotic activity, hypercellularity, and the infiltration of skeletal muscle. Notably, all patients with perineuriomas showing these atypical features have experienced a benign clinical course [63].

#### 7.2.1. Immunohistochemical Features

Like intraneural perineurioma, soft tissue perineurioma is stained with EMA and GLUT1; variable immunoreactivity has been documented for CD34, α-smooth muscle actin, and pancytokeratins [1].

#### 7.2.2. Molecular Features

Although not associated with NF1 or any type of schwannomatosis, soft tissue perineuriomas share molecular alterations with other nerve sheath tumors, including *NF2* point mutations and deletions of chromosome 13 and 22q12 (NF2) and the deletion of 17q11 (including NF1) [1,51]. Interestingly, *TRAF7* mutations, seen in intraneural perineuriomas, are not observed in the soft tissue counterpart [52].

## 8. Hybrid Tumors

**Figure 20:**
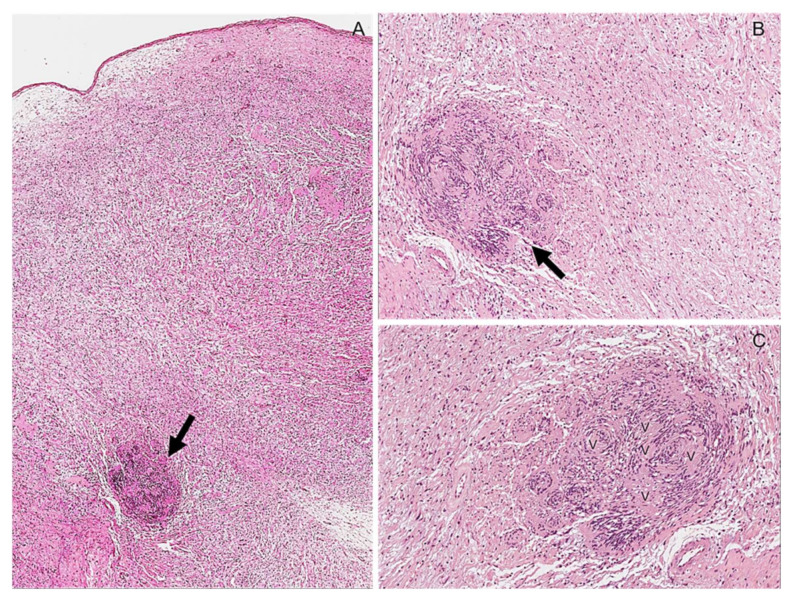
Hybrid neurofibroma/schwannoma tumor. (**A**) Low-magnification image showing a hypocellular tumor that predominantly exhibits a conventional neurofibroma morphology, along with small nodules reminiscent of schwannoma (arrow). (**B**) Higher magnification image better showing the nodule of schwannoma (arrow). (**C**) Verocay bodies (v) are seen within the schwannoma nodule.

Although most peripheral nerve sheath tumors can be easily diagnosed by surgical pathologists and are correctly classified as neurofibromas, schwannomas, and perineuriomas, a minority of neoplasms are difficult to categorize into one specific diagnostic category. Most of these tumors exhibit a plexiform architecture and are represented by a variable combination of neurofibromatous and schwannomatous areas (hybrid tumors) (Figure 20). Most tumors are associated with the different types of schwannomatosis and less frequently with NF1 [64]. It has been suggested that the hybrid tumors arising in the context of NF1 patients may be better defined as “*plexiform neurofibromas with schwannoma-like nodular proliferations*” [1]. Notably, about 45% of these tumors have been shown to be associated with chromosome 22 monosomy [65,66]. Apart from hybrid tumors with neurofibroma/schwannoma features, there is increasing evidence of a distinct category composed of a mixture of perineurioma and schwannoma areas [1,67,68]. Although histologically similar to soft tissue perineurioma, such tumors are composed of two different cytotypes, namely, perineurial cells (EMA/claudin/GLUT1-positive) variably arranged into fascicular, storiform, or reticular arrangements and spindled cells (S100/SOX10-positive) with wavy nuclei, closely reminiscent of schwannian differentiation. Only rarely, hybrid tumors with both neurofibroma and perineurioma features have been reported in the literature [69,70,71,72]. Their classification is still to be defined, as it is not clear if they represent a “true” distinct category or simply neurofibromas rich in perineurial cells.

### Molecular Features

Most cases of hybrid schwannoma/perineuriomas displayed VGLL3 rearrangements [69,70].

## 9. Malignant Peripheral Nerve Sheath Tumors (MPNSTs)

MPNSTs represent about 5% of all sarcomas and may arise from a peripheral nerve or a pre-existing neurofibroma; in about 50% of cases, these tumors arise in the context of NF1, while in the remaining cases they appear to be sporadic (40% of cases) or associated with a previous history of radiation [1,4,6,73,74,75]. A significant number of the sporadic tumors arise in the deep soft tissues without any anatomic evidence of association with a peripheral nerve or pre-existing neurofibroma (soft tissue MPNST) [73,74,75]. The sporadic tumors usually occur in patients with an age ranging from 30 to 50 years, while the NF1-asociated tumors can manifest in younger patients, including in childhood [73,74,75]. The most common sites are the extremities, trunk, and head/neck region; among the major nerves involved, the sciatic nerve is the most common one, followed by the brachial and sacral plexuses and the paraspinal nerves. Clinically MPNSTs present as growing painless or painful masses; in NF1 patients, any rapid enlargement of a pre-existing neurofibroma is suggestive of malignant transformation. Grossly, tumor masses are large-sized (usually >5 cm) with a white-gray firm to fleshy cut surface; necrotic areas and hemorrhage are common. Histologically, the following subtypes of MPNSTs are recognized (Table 15): (i) classic MPNSTs; (ii) epithelioid cell MPNSTs; (iii) perineural MPNSTs (so-called malignant perineurioma); and (iv) malignant schwannian melanotic tumors (Table 16) [1].

### 9.1. Classic MPNST

**Figure 21:**
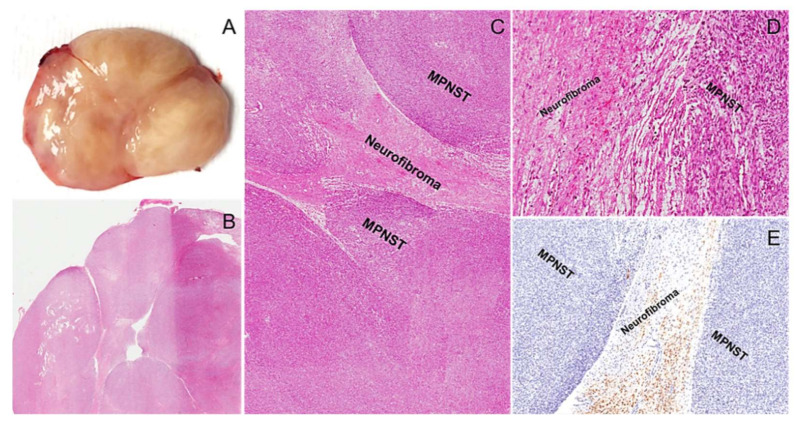
Classic malignant peripheral nerve sheath tumor (MPNST) arising in a neurofibroma. (**A**) Grossly, the tumor exhibits a multinodular, yellow to whitish in color, appearance. (**B**) Low-magnification image showing a hypercellular spindle cell tumor with a multinodular architecture. (**C**) Areas of low-grade MPNST are intermingled with a residual neurofibroma component. (**D**) Abrupt transition of residual neurofibroma into low-grade MPNST is seen. (**E**) Unlike MPNST, neurofibroma cells are strongly and diffusely stained with S100 protein.

**Figure 22:**
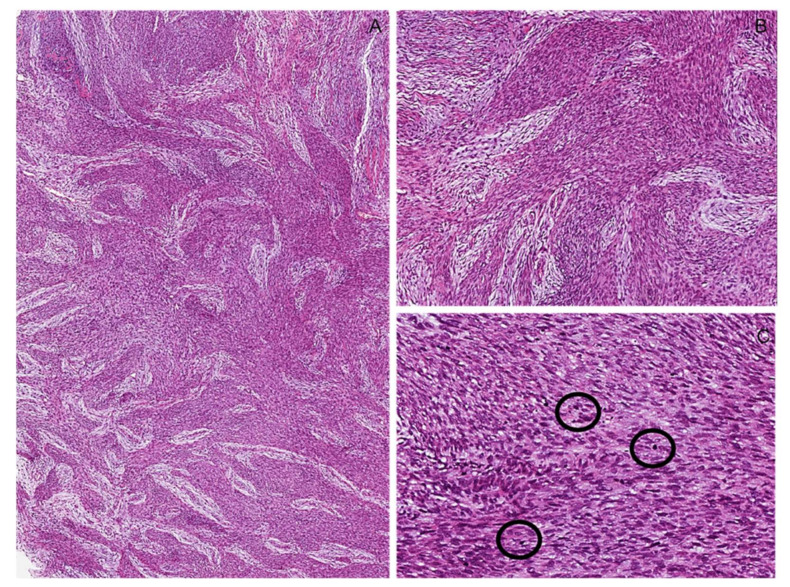
High-grade classic malignant peripheral nerve sheath tumor (MPNST). (**A**,**B**) Low- and medium-magnification images showing a hypercellular tumor composed of uniform spindled cells arranged in densely cellular fascicles, often alternating and interdigitating with more hypocellular and myxoid areas (*marble-like* appearance). (**C**) Numerous mitoses (circles) are seen in high-grade MPNSTs.

**Figure 23:**
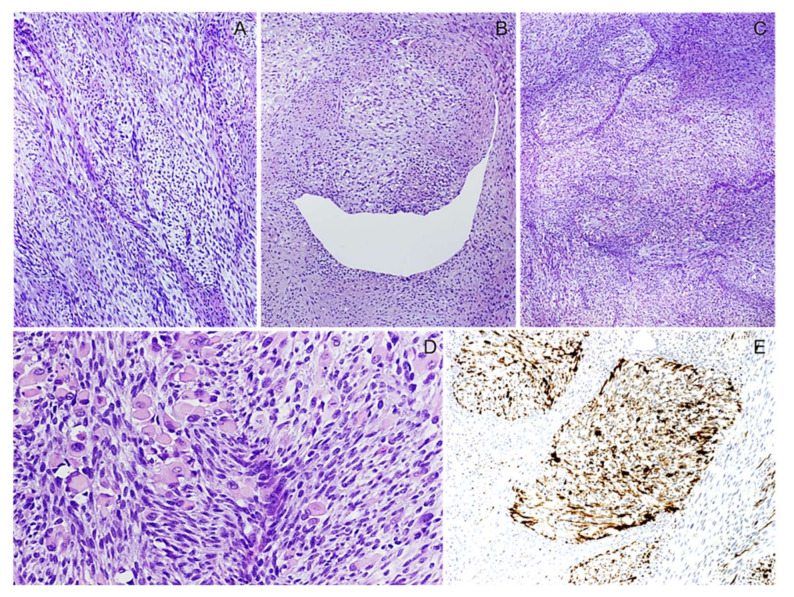
Triton Tumor. (**A**) Low-magnification image showing a spindle cell sarcoma with a fascicular growth pattern. (**B**) Perivascular condensation of neoplastic cells herniating into the vascular lumens is seen. (**C**) Notably, the tumor contains nodules that, at a higher magnification (**D**), are referred to as a heterologous mesenchymal component consisting of rhabdomyoblasts. (**E**) Rhabdomyoblasts are diffusely stained with desmin.

The most common subtype of MPNSTs is the classic type, basically a spindle cell sarcoma that can be divided into low- and high-grades based on the following morphological features: (i) the loss of neurofibroma architecture; (ii) hypercellularity; (iii) cytological atypia; (iv) mitotic activity; and (v) tumor necrosis [1,73,74,75]. Low-grade MPNSTs are uncommon (10% of all MPNSTs) and usually arise from a pre-existing NF1-associated neurofibroma (Figure 21) [1,76]. Although morphologically similar to atypical neurofibromatous neoplasms with uncertain biologic potential (ANNUBP), they differ in that mitotic activity ranges from >3 to 9 mitoses/10 HPF; tumor necrosis is absent by definition [6]. Accordingly, before making a diagnosis of atypical neurofibromatous neoplasm with uncertain biologic potential (ANNUBP) (>1 mitosis/50 HPF but <3 mitoses/10 HPF), an extensive sampling of the tumor and a meticulous search of mitoses are necessary to rule out the possibility of a low-grade MPNST [6]. Most MPNSTs (85–90%) are high-grade spindle cell sarcomas with diffuse moderate-to-severe cytological atypia, high mitotic activity, and tumor necrosis. A high grade is equally assigned to tumors in the presence of both tumor necrosis and mitotic activity ranging from 3 to 9 mitoses/10 HPF or if mitotic activity is >10 HPF, even in the absence of tumor necrosis [6]. MPNSTs are usually composed of relatively uniform spindled cells arranged in densely cellular fascicles and/or whorls (fibrosarcoma-like appearance), often alternating and interdigitating with more hypocellular and myxoid areas (marble-like appearance) (Figure 22). Some tumors may exhibit, at least focally, other growth patterns, including herringbone, nodular, curlicue, and palisading patterns. The neoplastic cells usually have pale eosinophilic cytoplasm with ill-defined cellular borders and hyperchromatic elongated nuclei. The tumor stroma is variably fibro-myxoid in nature. Although not pathognomonic, in most cases there is a perivascular condensation/accentuation of neoplastic cells that appear to push or herniate into the vascular lumens. Extensive geographic tumor necrosis with a peritheliomatous pattern (viability of neoplastic cells limited to perivascular regions) is a common feature [1]. Unusual features include the presence of collagen bands and/or nodules (collagen rosettes), focal epithelioid or, more rarely, small cell morphology (likely neuroepithelial differentiation) [1]. In a minority of cases, MPNSTs may contain areas of marked nuclear anaplasia closely reminiscent of undifferentiated pleomorphic sarcoma [1]. Notably, a small subset of MPNSTs (10% of cases), especially those arising in NF1 patients, may contain cellular lines of divergent differentiation (heterologous elements), including a variable number of relatively mature rhabdomyoblasts (so-called “Triton tumor”) (Figure 23) and osteosarcomatous, chondrosarcomatous, and more rarely, angiosarcomatous or liposarcomatous components; well-differentiated glands, occasionally with malignant cytology, composed of cuboidal/columnar clear cells can be exceptionally appreciated [1,77,78,79]. The 5-year survival rate is 51%, and the prognosis seems to be closely related to radical surgery (10-year survival: 80% versus 14%, respectively, for patients who had radical or incomplete surgery) [1,6,14,76].

#### Immunohistochemical Features

Immunohistochemically, a diffuse staining for S100 protein and/or SOX10 is a rare feature, with only 40% of cases showing only focal expression [1]. The loss of trimethylated histone 3 at lysine residue 27 (H3K27me3) expression due to the inactivation of polycomb repressor complex (PRC2) for mutations of the *SUZ12* gene is a diagnostic tool [1,5,80]. As a mosaic expression pattern of H3K27me3 (alternating of positive and negative cells) can be obtained in other sarcomas, especially synovial sarcoma, with which MPNST shares some morphological features, only a complete loss of H3K27me3 expression should be considered useful for the diagnosis of MPNSTs in the appropriate clinicopathologic context [1].

### 9.2. Epithelioid Cell MPNST

**Figure 24:**
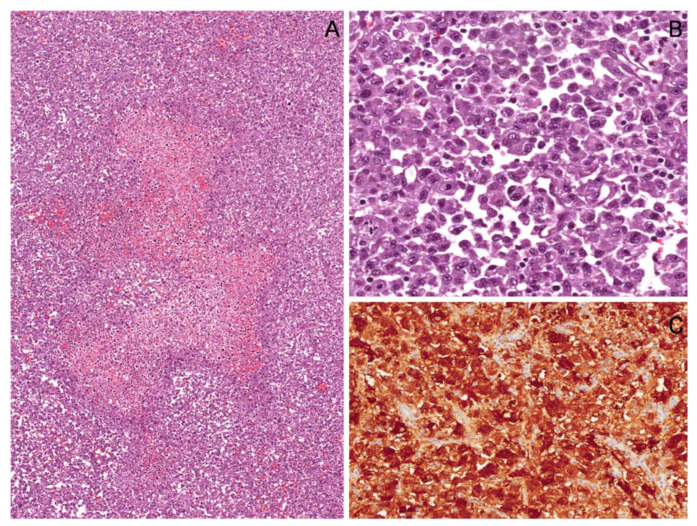
Epithelioid cell malignant peripheral nerve sheath tumor (MPNST). (**A**) Low-magnification image showing a hypercellular tumor with a central area of necrosis. (**B**) Tumor is composed of nests and/or cords of large-sized epithelioid cells with abundant eosinophilic to amphophilic cytoplasm and large rounded nuclei with vesicular chromatin and prominent nucleoli. (**C**) Unlike classic-type MPNST, epithelioid cell MPNST characteristically exhibits diffuse and strong immunoreactivity for S100 protein.

Epithelioid cell malignant peripheral nerve sheath tumor (MPNST) is a rare subtype, accounting for approximately 5% of all MPNSTs [1,4,81,82,83,84,85]. By definition, these tumors are composed predominantly or exclusively of Schwann cells with a polygonal/epithelioid morphology. Their incidence and age distribution mimic those of its more common spindle cell counterpart, with most cases occurring mainly in adults (40–50 years) with a slight male predominance. Epithelioid cell MPNSTs arise sporadically in the deep soft tissues from major nerves of the extremities or trunk, including the sciatic, tibial, peroneal, facial, antebrachial cutaneous, and digital nerves; occasionally some cases have been documented to occur in superficial soft tissues or in the skin. Only a few cases have been documented in the context of NF1 patients [81]. Clinically, they present as slowly growing painful or painless masses. Notably, a significant number of epithelioid cell MPNSTs develop from pre-existing sporadic schwannomas, often exhibiting an epithelioid cell morphology, and they develop only exceptionally in patients with schwannomatosis [1]. Grossly, they present as well-demarcated multinodular masses that are firm in consistency and have a fleshy greyish cut surface. Histological examination shows a tumor with a multinodular growth pattern composed of nests and/or cords of large-sized epithelioid cells with abundant eosinophilic to amphophilic cytoplasm and large rounded nuclei with vesicular chromatin and prominent nucleoli (Figure 24). Cellularity is variable, ranging from densely cellular tumors to hypocellular myxoid lesions. The tumor stroma is variably fibro-myxoid. Mitoses and necrosis are relatively common. Although most tumors are composed exclusively/predominantly of epithelioid cells, a minor spindle cell component closely resembling a classic MPNST can be occasionally seen. Unusual histologic features, including clear cell changes and a rhabdoid cell morphology, have been reported. Due to their morphological appearance, differential diagnosis with metastatic epithelioid cell melanoma, undifferentiated carcinoma, or primary or metastatic proximal-type epithelioid cell sarcoma is extremely difficult on morphology alone unless an origin from a nerve or schwannoma can be demonstrated. Epithelioid MPNSTs are malignant lesions with significant metastatic potential, usually to the lungs. It is still to be established if superficially located tumors have a better prognosis than deep-seated ones [1].

#### Immunohistochemical Features

Unlike classic MPNSTs, epithelioid cell MPNSTs typically show a diffuse and strong immunohistochemical expression of S100 protein and SOX-10 [1]; in addition, the loss of nuclear expression of SMARCB1 (INI-1) has been documented in approximately 50% of cases [84], while the nuclear expression of H3K27me3 is typically retained [1]. Unlike in melanoma, Melan-A, MITF, and HMB-45 are not expressed. Although cytokeratin expression can be occasionally documented, posing differential diagnostic problems with metastatic carcinoma, the latter lacks S100 protein and SOX10 expression [1].

### 9.3. Perineurial Malignant Peripheral Nerve Sheath Tumors (Malignant Perineurioma)

Malignant perineurioma, also called “*Malignant Peripheral Nerve Sheath Tumor with perineurial differentiation*” represents an extremely rare variant of MPNST that is not related to NF1 or schwannomatoses (Table 16) [1,85,86,87]. The most commonly reported sites of occurrence include the extremities, trunk, and face as well as visceral sites, the mediastinum, and the retroperitoneum of adult patients. These tumors are distinguished from their benign counterpart by the presence of severe nuclear atypia, hypercellularity, and high mitotic activity. Malignant perineurioma has been further classified, on the basis of malignant histological features, into low-grade and high-grade perineurial MPNST. Low-grade tumors are histologically reminiscent of soft tissue perineurioma (so-called “*perineural sarcoma*”) from which they differ for worrisome features, such as infiltrative growth, areas of increased cellularity, cytologic atypia, and mitotic figures [1]; tumor necrosis is lacking. Immunohistochemically, they express markers consistent with perineurial differentiation (EMA, claudin-1, and GLUT1). On the other hand, high-grade perineurial MPNST is a pleomorphic spindle cell sarcoma showing prominent cytologic atypia and numerous mitotic figures. These tumors lack obvious morphological features of perineurioma. Therefore, an extensive sampling of these neoplasms and a careful morphologic evaluation are required to detect, at least focally, features suggestive of perineurial differentiation, such as whorls and/or cells with delicate overlapping cell processes. Moreover, positivity for EMA, claudin-1, and/or GLUT1 is detected, at least focally. Given their rarity, the biologic behavior of these tumors is still unclear. To date, malignant perineuriomas, despite their potential for distant metastases and local recurrences, are believed to be less aggressive when compared to conventional malignant peripheral nerve sheath tumors. The most challenging differential diagnosis for low-grade malignant tumors is represented by low-grade fibromyxoid sarcoma, as this tumor may show similar morphology as well as immunohistochemical expression of EMA and claudin-1. However, the immunohistochemistry for MUC4 and the FISH analysis for *FUS* rearrangement are distinctive features of low-grade fibromyxoid sarcoma that were never observed in perineurial tumors.

## 10. Malignant Melanotic Schwannian Tumor (So-Called *“Melanotic Schwannoma”*)

**Figure 25:**
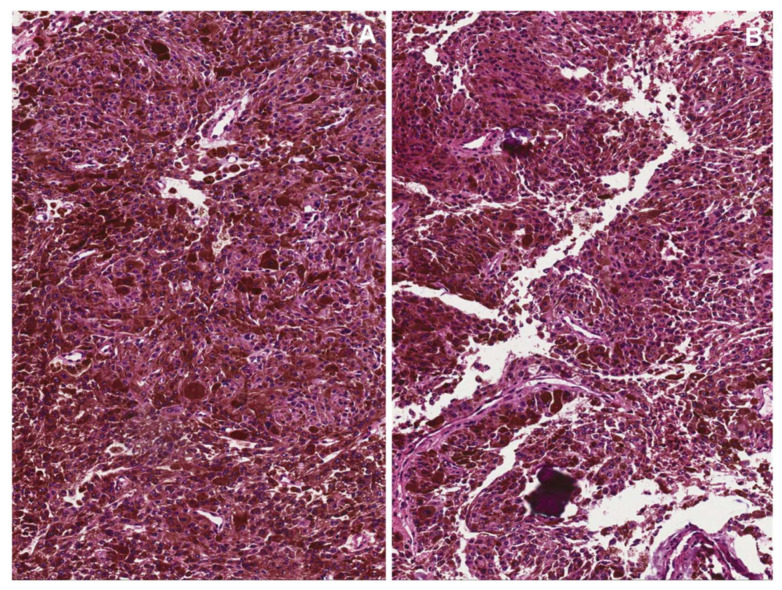
Malignant melanotic nerve sheath tumor. (**A**) Tumor is composed of heavily pigmented plump spindled to polygonal cells arranged in interlacing fascicles or nests. (**B**) The presence of psammoma bodies (arrows) is a characteristic feature of this tumor.

Formerly known as *“melanotic schwannoma”*, malignant melanotic schwannian tumor is a neoplasm with an uncertain risk for aggressive behavior, usually arising from the spinal or autonomic nerves near the midline [1,88,89,90,91]. Although sporadic and in most cases solitary, this tumor can occur in <5% of patients with Carney complex, and about 20% of these patients may present with multiple lesions [91]. Clinical symptoms, such pain and neurologic manifestations, are related to the tumor location. Grossly, the tumors are circumscribed and variably encapsulated and characteristically show a black-brown cut surface. Histologically, tumors are composed of relatively bland-looking spindled to polygonal cells arranged in fascicles; less commonly, nuclear palisading and/or whorls may be detected, suggesting schwannian differentiation. They characteristically contain heavy deposits of intracytoplasmic melanin pigment, while nuclei often show clear pseudoinclusions and marked nuclear pleomorphisms (hyperchromatism and/or nucleomegaly) as well as prominent nucleoli (Figure 25). Notably, numerous psammoma bodies are present in most cases. Although tumors with >2 mitoses/10 HPF may have a metastatic risk, the clinical behavior of malignant melanotic schwannian tumor is uncertain in that metastases may occur even in the absence of adverse morphological features. As metastases have been variably reported in 26–53% of cases [90,92,93], the definition of “*tumor with uncertain biological behavior*” seems to be appropriate [1].

### Molecular Features

Molecular studies have identified *PRKAR1A* mutations in most cases, despite their occurrence in the context or without Carney complex [91,92].

## 11. Conclusions

Peripheral nerve sheath tumors are relatively common lesions that exhibit a wide morphological and biological spectrum. In the presence of conventional morphological and immunohistochemical features, the histological diagnosis is usually straightforward, but they may represent diagnostic challenges. As these neoplasms may arise in the context of tumor-predisposing syndromes, such as NF1 and the different types of schwannomatosis, it is crucial for surgical pathologists to provide a correct diagnosis in order to plan the appropriate clinical management of the patients.

## Figures and Tables

**Table 1:** Classification of the peripheral nerve sheath tumors.

**Benign tumors**
▪ Neurofibromas ▪ Schwannomas ▪ Perineuriomas ▪ Hybrid tumors
**Tumors with uncertain malignant potential**
▪ Atypical neurofibromatous neoplasm with uncertain biologic potential (ANNUBP)” (in NF1 patients)
**Malignant tumors**
▪ Classic malignant peripheral nerve sheath tumors ▪ Epithelioid cell malignant peripheral nerve sheath tumors ▪ Perineural malignant peripheral nerve sheath tumors

**Table 2:** Neurofibromatosis type 1 and related/alternative disorders: clinical phenotypes and genotypes.

**Neurofibromatosis type 1** [NF1; MIM # 162200; ORPHA:636] Chromosome 17q11.2 (*NF1* gene): heterozygous pathogenic *NF1* gene variant in unaffected tissues (e.g., white blood cells) *; *Major features*: multiple large [>5 mm in pre-pubertal children vs. >1.5 cm in post-pubertal children/adults] and/or small (i.e., the so-called “freckling” in specific places including axillae, groin, peri-oral, sub-mammary) café-au-lait spots * in the skin; two or more iris Lisch nodules (by slit lamp examination) *; two or more eye choroidal abnormalities (i.e., bright, patchy nodules detected by optical coherence tomography, OCT or near-infrared reflectance, NIR) *; one or more neurofibromas (cutaneous, sub-cutaneous/nodular, internal) *; *Minor features*: macrocephaly/megalencephaly (98th centile); delayed growth (stature 10–25th centile); congenital thoracic deformities; dysmorphic features; *Complications*: anterolateral long bone dysplasia (bowing) and/or pseudarthrosis *; (kypho) scoliosis; sphenoid wing dysplasia *; osteopenia/porosis; puberty disorders; hypertension; congenital heart defects (CHD); vasculopathy; haemostasis defects; optic pathway glioma (OPG) *; plexiform neurofibroma (PNF) *; learning difficulties; brain high (bright) signal lesions on T2 images (FASI); brainstem gliomas (BSG); increased risk of developing neoplasia [e.g., malignant peripheral nervous sheath tumour (MPNST), breast cancer, phaechromocytoma, neuroblastoma, gastrointestinal neuroendocrine tumour (GIST). NF1 Microdeletion syndrome [ORPHA:97685]: type 1 (1.4 Mb), type 2 (1.2 Mb), type 3 (1.0 Mb), atypical (> 1.4 Mb?): dysmorphisms, congenital heart defects, intellectual disability, neurofibromas (early-onset), sphenoid wing dysplasia, increased frequency of MPNSTs.
**Spinal neurofibromatosis type 1** [MIM # 162210] Chromosome 17q11.2 (*NF1* gene): heterozygous pathogenic *NF1* gene variant [missense variants] in unaffected tissues (e.g., white blood cells) or in affected tissues (e.g., neurofibromas); *Main features*: multiple neurofibromas in all (38) spinal roots in the absence (or with few) classical/typical NF1stigmata (see above)
**Mosaic (segmental) neurofibromatosis type 1** [MIM # 162210] Chromosome 17q11.2 (*NF1* gene): (**a**) a pathogenic heterozygous *NF1* gene variant in a clearly affected tissue with the typical NF1 stigmata [see below] in the absence of a pathogenic heterozygous *NF1* gene variant in unaffected tissue (e.g., blood or other body areas); (**b**) an identical pathogenic heterozygous *NF1* gene variant in two anatomically independent affected tissues with the typical NF1 stigmata, in the absence of a pathogenic heterozygous *NF1* gene variant in unaffected tissue; or (**c**) a heterozygous *NF1* gene variant with a variant allele fraction of significantly less than 50% in apparently normal tissue (e.g., blood); *Main features*: NF1 stigmata clearly distributed in a segmental/localised (mosaic) region/pattern: (**1**) Skin pigmentary manifestations only; (**2**) Neurofibromas only; (**3**) Skin pigmentary manifestations and neurofibromas (rare); (**4**) Plexiform neurofibroma alone; (**5**) Unilateral iris Lisch nodules; (**6**) a parent, with a child fulfilling the criteria for NF1, who has only one NF1 diagnostic criterion including small café-au-lait spots (i.e., freckling) in specific places (i.e., axillae, groin, peri-oral, sub-mammary), optic pathway glioma, two or more iris Lisch nodules or two or more choroid abnormalities, distinctive osseous lesion for NF1, two neurofibromas or a plexiform neurofibroma; *Complications*: decreased frequency of typical NF1 complications (<7%)
**Watson syndrome** [MIM # 193520] Chromosome 17q11.2 (NF1 gene) *Main features*: pulmonic stenosis, multiple café-au-lait spots, decreased intellectual ability, short stature; this NF1 alternative form is disputed, however, clinicians and pathologists should be aware that individuals harbouring pathogenic *NF1* gene variants could manifest these features.
**Neurofibromatosis/Noona syndrome** [MIM # 601321; ORPHA:638] Chromosome 17q11.2 (*NF1* gene) Chromosome 12q24.13 (*PTPN11* gene) Main features: typical NF1 features + Noonan syndrome features [i.e., facial dysmorphic features (large forehead, hypertelorism, ptosis, down-slanting ocular rims, low-set/dysmorphic ears); webbed neck; congenital heart defects; thoracic deformities; learning difficulties; this NF1 alternative form is disputed, however, clinicians and pathologists should be aware that individuals harbouring pathogenic *NF1/PTPN11* gene variants could manifest these features.
**Constitutional mismatch repair cancer syndrome** [MMRCS; MIM # 276300] Including: Turcot and Lynch [HNPCC1] syndromes or hereditary non-poliposis colo-rectal cancer (HNPCC) syndrome. Chromosome 2p21p16 (*MSH2* gene); 2p16.3 (*MSH6* gene); 3p22.2 (*MLH1* gene); 7p22.1: biallelic DNA mismatch repair pathogenic variants. *Main features*: NF1-like macules and/or peripheral nerve tumours and malignant gliomas.

* = Criterion included within the diagnostic criteria for NF1 [2].

**Table 3:** Key diagnostic features of isolated cutaneous/nodular neurofibromas.

**Definition**
benign peripheral nerve sheath tumor mainly composed of Schwann cells with nodular architecture
**Clinical Features**
young to middle-aged adults **usually solitary, often not associated to other NF1 stigmata** less frequently associated with NF-1 predilection for the skin of trunk, head, and neck region and extremities less frequently located at deep nerves **painless nodules (when nodular and located in deep nerves may cause pain afetr compression)** sensory or motor deficit in intraneural tumors
**Gross Pathology**
well-circumscribed dermal/subcutaneous nodule with glistening tan-white cut section encapsulated, fusiform-shaped mass when a major nerve is affected
**Histopathology**
low to moderate cellularity bland-looking spindle cells with small wavy or comma-shaped nuclei and poorly defined cytoplasm haphazard cell arrangement variably fibro-myxoid stroma with collagen fibrils and ropey collagen bundles
**Immunohistochemistry**
diffuse expression of S-100 protein and SOX-10 variable expression of EMA and CD34
**Treatment/Prognosis**
benign tumor surgical excision with free margins is usually curative malignant transformation (classic malignant peripheral nerve sheath tumor) is unlikely for cutaneous tumors intraneural tumors, especially if NF-1-associated, may represent the morphological precursor of MPNST

**Table 4:** Key diagnostic features of diffuse neurofibromas.

**Definition**
benign peripheral nerve sheath tumor mainly composed of Schwann cells with diffuse growth pattern
**Clinical Features**
younger age group than nodular neurofibroma **usually solitary (sometimes not associated to other NF1 stigmata)** **should be regarded as mosaic NF1 manifestations; other NF1 stigmata should be looked for** plaque-like lesion located on the skin of the head and neck region
**Gross Pathology**
ill-defined dermal/subcutaneous nodule with tan-white cut section
**Histopathology**
low to moderate cellularity bland-looking spindled cells with small hyperchromatic and wavy or comma-shaped nuclei haphazard cell arrangement fine fibrillary to myxoid stroma pseudomeissnerian-body-like structures infiltration of the subcutaneous adipose tissue with, at least focally, a honey-comb pattern large ectatic and focal branching capillary-like vessels in NF-1 patients
**Immunohistochemistry**
diffuse expression of S-100 protein and SOX-10 variable expression of EMA and CD34
**Treatment/Prognosis**
benign tumor surgical excision with free margins is usually curative malignant transformation to malignant peripheral nerve sheath tumor is unlikely

**Table 5:** Key diagnostic features of plexiform neurofibroma.

**Definition**
benign peripheral nerve sheath tumor composed mainly of Schwann cells with multinodular/plexiform architecture
**Clinical Features**
childhood tumor NF1-associated tumor (virtually pathognomonic of NF1); **when truly isolated/solitary think should be regarded as mosaic NF1** frequent origin from the large nerve trunks of the head and neck region, trunk, or extremities
**Gross Pathology**
multinodular mass with “*bag of worms appearance*”, consisting of serpentine structures reminiscent of intermingling nerve fascicles
**Histopathology**
low to moderate cellularity bland-looking rounded to spindled cells with hyperchromatic wavy or comma-shaped nuclei multinodular and serpentine nerve-like structures abundant myxo-edematous stroma with thick haphazardly arranged collagen fibers (*shredded carrots appearance*) occasional extension to the surrounding tissues, closely resembling diffuse neurofibroma
**Immunohistochemistry**
diffuse expression of S-100 protein and SOX-10 variable expression of EMA and CD34
**Treatment/Prognosis**
surgical excision with free margins is usually curative highest risk among all neurofibroma types of malignant transformation to malignant peripheral nerve sheath tumor

**Table 6:** Atypical neurofibromatous neoplasm with uncertain biologic potential (ANNUBP).

**Definition**
Provisional diagnostic category of neurofibromatous tumor in NF1 patients
**Histological diagnosis (presence of at least two of the following features)**
nuclear atypia hypercellularity increased mitotic activity (>1 mitosis/50 HPF but <3 mitoses/10 HPF) variable loss of neurofibroma architecture (i.e, the presence of herringbone and/or fascicular storiform growth patterns)

**Table 7:** NF2/SMARCB1/LZTR1-related schwannomatoses (schwannoma predisposing syndromes).

**NF2-related schwannomatosis** **[NF2/MERLIN-schwannoma predisposing syndrome (NF2/MERLIN-SPS)]** (Previously, **neurofibromatosis type 2** or **NF2**) [MIM # 101000; ORPHA:637] Chromosome 22q12.2 (*NF2/MERLIN* moesin-ezrin-radixin-like gene): identical *NF2* gene pathogenic variant in at least two anatomically distinctNF2related tumours (e.g., schwannoma, meningioma and/or ependymoma); NF2 gene variants in unaffected tissues (e.g., blood) and major/minor criteria (see below); *Main features*: (**1**) *Gardner type* (adulthood): Bilateral (or, sometimes, unilateral) VIII cranial nerve (vestibular) schwannoma(s) *; schwannomas of cranial nerves *; multiple meningiomas, ependymomas, schwannomas *; early-onset (posterior subcapsular or cortical) cataracts *; skin schwannomas (NF2 plaques) *; nodular schwannomas *; (**2**) *Wishart* (severe) *type* (childhood): prior to appearance of VIII nerve schwannomas * and/or nervous system tumours (meningiomas, ependymomas) *, non-VIII-cranial nerve schwannomas (e.g., mixed nerves, V, VII) *; early-onset (posterior subcapsular or cortical) cataract *; epiretinal membranes/hamartomas *; skin schwannomas (NF2 plaques) * diffused over body; brain cortical dysplasia; bone dysplasia; (3) *Congenital type* (neonatal/< 1 year): small bilateral VIII nerve schwannomas * stable for decade(s); optic nerve sheath meningioma(s) *; epiretinal membranes/hamartomas *; early-onset (posterior subcapsular or cortical) cataract *; skin schwannomas (NF2 plaques) * in atypical places (face, arms, legs) later disappearing; ependymomas *; spinal cord schwannomas and meningiomas *; brain cortical dysplasia;
**Mosaic (segmental)*****NF2*****-related schwannomatosis** **[Mosaic NF2/MERLIN-schwannoma predisposing syndrome]** **(Mosaic NF2/MERLIN-SPS)** (Previously, **mosaic neurofibromatosis type 2** or **mosaic NF2**) [MIM # 101000; ORPHA:637] Chromosome 22q12.2 (*NF2/MERLIN* moesin-ezrin-radixin-like gene): NF2 gene pathogenic variant (variant allele fraction) in unaffected tissue (e.g., blood) < 50% *Main features*: NF2 stigmata distributed in a segmental/localised (mosaic) distribution(e.g., unilateral VIII nerve schwannoma, ipsilateral meningiomas, schwannomas); Diagnosis ➔ NF2 gene pathogenic variant in unaffected tissue (e.g., blood) < 50%
***SMARCB1*****-related schwannomatosis (SWNTS 1)** ***LZTR1*****-related schwannomatosis (SWNTS2)** **[SMARCB1/LZTR1-schwannoma predisposing syndrome (SMARCB1/LZTR1-SPS)]** [SWNTS1, MIM # 162091; SWNTS2, MIM # 615670] Chromosome 22q11.23 (*SMARCB1* gene); chromosome 22q11.21 (*LZTR1* gene): (a) at least one pathologically confirmed schwannoma or hybrid nerve sheath tumour and *SMARCB1* or *LZTR1* pathogenic variant in an unaffected tissue (e.g., blood); (b) a common *SMARCB1* or *LZTR1* variant in two anatomically distinct tumours; **Main features**: multiple non-VIII cranial nerve, non-intradermal, cranial, spinal and peripheral schwannomas (in the absence of NF2 stigmata); *SWNTS1* = additional extra-axial, extra-medullary meningiomas and occasionally unilateral VIII nerve schwannomas; *SWNTS2* = later onset of disease (up to 60 years), schwannomas affecting various body regions (extremities, spinal cord, chest wall, subcutaneous);
**Mosaic*SMARCB1*-related schwannomatosis (SWNTS 1)** **Mosaic*****LZTR1*****-related schwannomatosis (SWNTS2)** **[Mosaic SMARCB1/LZTR1-schwannoma predisposing syndrome]** **(Mosaic SMARCB1/LZTR1-SPS)** [Mosaic SWNTS1, MIM # 162091; Mosaic SWNTS2, MIM # 615670] Chromosome 22q11.23 (*SMARCB1* gene); chromosome 22q11.21 (LZTR1 gene): (a) at least one pathologically confirmed and SMARCB1 or *LZTR1* pathogenic variant in an unaffected tissue (e.g., blood) in < 50% cells analysed; (b) a common *SMARCB1* or *LZTR1* variant in two anatomically distinct tumours *Main features*: see the above features of above SWNTS1 and SWNTS2
**22q-related-scwhanommatosis** **[22q-Schwannoma predisposing syndrome (22q-SPS)]** Chromosome 22q12.2 (*NF2/MERLIN* moesin-ezrin-radixin-like gene): Main features: patients who do NOT meet criteria for *NF2/MERLIN*-related-schwannomatosis, *SMARCB1*-related-schwanomatosis (SWNTS1) or *LZTR1*-related-scwhannomatosis (SWNTS2) but have both: (1) LOH of the same chromosome 22q markers in two anatomically distinct tumours (e.g., schwannoma or hybrid nerve sheath tumour); (2) A different NF2 pathogenic variant in each tumour but not in the unaffected tissue.

* = Criterion included within the proposed diagnostic criteria for NF2-related-schwannomatosis (schwannoma predisposing syndrome).

**Table 8:** Key diagnostic features of classic-type schwannoma.

**Definition**
encapsulated benign peripheral nerve sheath tumor composed of Schwann cells
**Clinical Features**
young to middle-aged adults **but also children [see “Wishart and Congenital types of NF2-related-schwannomatosis] (Table 2)** **often isolated/solitary** **within the context of the different types of schwannomatoses (Table 2)** exceptionally rare in NF-1 patients slow-growing, painless mass painful mass in large-sized nerve
**Gross Pathology**
encapsulated, fusiform-shaped mass when a small nerve is affected eccentric mass in large-sized nerve pinkish-white in color with frequent hemorrhagic foci and/or cystic degeneration on cut section
**Histopathology**
alternating hypercellular (Antoni A) and hypocellular (Antoni B) areas Antoni A areas: compact spindle cells with wavy nuclei, arranged in short, whorling, and/or intersecting fascicles; nuclear palisading (Verocay bodies) Antoni B areas: hypocellular areas of spindled to ovoid cells haphazardly arranged and set in a loose myxoid stroma containing microcystic changes, inflammatory cells, collagen fibers, and numerous thick-walled, large-sized vessels with perivascular hyalinization Mitoses: rare to absent Peripheral lymphocytic rim in tumors arising in the gastrointestinal tract
**Immunohistochemistry**
diffuse expression of S-100 protein and SOX-10 occasional aberrant expression of cytokeratins, desmin, and TTF-1
**Treatment/Prognosis**
benign tumor surgical excision with free margins usually curative malignant transformation: very rare

**Table 9:** Key diagnostic features of schwannoma with degenerative/ancient changes (“*ancient schwannoma*”).

**Definition**
Histological definition: schwannoma with degenerative-type nuclear atypia and stromal changes
**Clinical Features**
usually large-sized and long-standing mass frequently deeply located tumors (especially the retroperitoneum and head and neck region)
**Gross Pathology**
pinkish-white in color with frequent hemorrhagic foci and/or cystic degeneration on cut section
**Histopathology**
moderate- to high-grade nuclear atypia of degenerative-type degenerative stromal changes, including micro/macrocystic degeneration, hemorrhages, calcifications, and stromal hyalinization frequent siderophages/histiocytes intermingled with neoplastic cells mitoses: absent to rare tumor necrosis: absent
**Immunohistochemistry**
similar to classic-type schwannoma
**Treatment/Prognosis**
benign tumor surgical excision with free margins is curative

**Table 10:** Key diagnostic features of cellular schwannoma.

**Definition**
schwannoma exclusively/predominantly composed of Antoni A areas and lacking Verocay bodies
**Clinical Features**
frequently deeply located tumors (especially in the retroperitoneum and posterior mediastinum) rarely located at the extremities
**Gross Pathology**
similar to that of classic-type schwannoma occasionally multinodular/plexiform architecture
**Histopathology**
exclusively/predominantly composed of hypercellular Antoni A areas Antoni B areas: absent or focal long sweeping fascicles arranged in a fascicular, herringbone, and/or storiform growth pattern mitotic activity: present (usually < 4–5 mitoses/10 HPFs) coagulative necrosis: up to 10% of cases infiltrative margins: may be present, resulting in bone erosion
**Immunohistochemistry**
diffuse expression of S-100 protein and SOX-10 retained nuclear expression of H3K27me3
**Treatment/Prognosis**
higher rate (4 to 40%) of local recurrence than the classic-type schwannoma when surgical excision is incomplete

**Table 11:** Key diagnostic features of plexiform schwannoma.

**Definition**
schwannoma with multinodular/plexiform architecture, often appreciated at gross examination
**Clinical Features**
**isolated/solitary lesions in most cases (think about mosaicism for the schwannomatoses genes)** **NF2- or SMARCB1/LZTR1-related schwannomatoses tumours in a minority of cases** frequently located on the skin of the head and neck region and the distal extremities rarely located in the deep soft tissues
**Gross Pathology**
multinodular/plexiform architecture usually encapsulated
**Histopathology**
multinodular/plexiform growth pattern predominantly composed of hypercellular Antoni A areas mitotes: absent to rare necrosis: absent cytological atypia: absent
**Immunohistochemistry**
diffuse expression of S-100 protein and SOX-10 retained nuclear expression of H3K27me3
**Treatment/Prognosis**
higher rate of local recurrence than the classic-type counterpart when surgical excision is incomplete

**Table 12:** Key diagnostic features of epithelioid cell schwannoma.

**Definition**
schwannoma composed exclusively/predominantly of epithelioid cells with schwannian differentiation
**Clinical Features**
sporadic in virtually all cases frequently located on the skin or superficial soft tissues of the extremities well-circumscribed and small-sized mass
**Gross Pathology**
similar to that of classic-type schwannoma
**Histopathology**
small to medium-sized rounded to epithelioid cells with abundant eosinophilic cytoplasm and rounded nuclei with prominent nucleoli and nuclear pseudoinclusions large deciduoid-like cells may be seen tumor cells arranged singly or in small nests and cords and set in a variably fibro-myxoid stroma collagen rosettes frequently present areas with conventional schwannoma morphology may be, at least focally, seen nuclear atypia and mitoses may be seen necrosis: absent
**Immunohistochemistry**
diffuse expression of S-100 protein and SOX-10 retained nuclear expression of H3K27me3 lack of SMARCB1/INI1 expression in up 40% of cases
**Treatment/Prognosis**
benign tumor surgical excision with free margins is usually curative very low risk of malignant transformation into epithelioid cell MPNST

**Table 13:** Key diagnostic features of intraneural perineurioma.

**Definition**
benign tumor composed of perineurial cells confined to the nerve fascicles
**Clinical Features**
children to young adults predilection for upper limbs and lower limbs segmental thickening of the affected nerve motor deficiency, muscle weakness, progressive loss of sensory function, and in rare cases, muscle atrophy
**Gross Pathology**
fusiform expansion of the affected nerve, extending several centimeters in length
**Histopathology**
proliferation of spindled cells with thin, elongated, eosinophilic, cytoplasmic processes arranged in concentric layers (*onion bulbs*) around preexisting Schwann cell–axon complexes
**Immunohistochemistry**
diffuse expression of EMA and variable expression of claudin-1 and GLUT-1 abnormalities and/or monosomy of the long arm of chromosome 22
**Molecular Diagnostic Features**
*TRAF7* mutations
**Treatment/Prognosis**
benign tumor no standard treatment guidelines complete resection may lead to variable loss of neural function

**Table 14:** Key diagnostic features of soft tissue perineurioma.

**Definition**
benign peripheral nerve sheath tumor composed of perineurial cells
**Clinical Features**
wide age range with a peak in middle-aged adults predilection for superficial soft tissues of the lower limbs, upper limbs, or trunk slow-growing painless nodule
**Gross Pathology**
well-circumscribed unencapsulated tumor
**Histopathology**
classic-type: slender fibroblast-like cells with long bipolar cytoplasmic processes variably arranged into a fascicular, storiform, whorled, or lamellar (Pacinian) growth pattern. reticular-type: slender spindled cells with cytoplasmic anastomosing processes resulting in a reticular or lace-like appearance; myxo-edematous stroma sclerosing type: spindled to rounded/epithelioid cells arranged in corded, trabecular, or whorled growth patterns; abundant fibro-sclerotic stroma
**Immunohistochemistry**
expression of EMA, claudin-1, and GLUT-1 variable expression of CD34
**Molecular Diagnostic Features**
deletion of 22q12, mutations in *NF2,* and deletion of 17q11
**Treatment/Prognosis**
benign tumor surgical resection with free margins is curative

**Table 15:** Classification of malignant peripheral nerve sheath tumors (MPNSTs).

Classic MPNSTs Epithelioid cell MPNSTs Perineurial MPNSTs Malignant melanotic schwannian tumors (so-called “*melanotic schwannoma*”)

**Table 16:** Key diagnostic features of perineural MPNST (malignant perineurioma).

**Definition**
extremely rare variant of MPNST, unrelated to NF1 or different types of schwannomatoses
**Clinical Features**
adult patients predilection for extremities, trunk, and face as well as visceral sites, mediastinum, and retroperitoneum
**Gross Pathology**
infiltrative margins
**Histopathology**
low-grade perineurial MPNSTs: infiltrative growth, hypercellularity, cytologic atypia, and occasional mitotic figures; no necrosis high-grade perineurial MPNST: pleomorphic spindle cell sarcoma showing prominent cytologic atypia, necrosis, and numerous mitoses
**Immunohistochemistry**
expression of EMA, claudin-1, and GLUT-1 variable expression of CD34
**Treatment/Prognosis**
Malignant tumor with the potential for local recurrences Low risk of distant metastases

## Data Availability

All data presented in this manuscript are available from the corresponding author upon reasonable request.
